# Arousal of Cancer-Associated Stroma: Overexpression of Palladin Activates Fibroblasts to Promote Tumor Invasion

**DOI:** 10.1371/journal.pone.0030219

**Published:** 2012-01-23

**Authors:** Teresa A. Brentnall, Lisa A. Lai, Joshua Coleman, Mary P. Bronner, Sheng Pan, Ru Chen

**Affiliations:** 1 GI Division, Department of Medicine, University of Washington, Seattle, Washington, United States of America; 2 Department of Anatomic Pathology, Cleveland Clinic Foundation, Cleveland, Ohio, United States of America; 3 Division of Anatomic Pathology, University of Utah, Salt Lake City, Utah, United States of America; University of Bergen, Norway

## Abstract

**Background:**

Cancer-associated fibroblasts, comprised of activated fibroblasts or myofibroblasts, are found in the stroma surrounding solid tumors. These myofibroblasts promote invasion and metastasis of cancer cells. Mechanisms regulating the activation of the fibroblasts and the initiation of invasive tumorigenesis are of great interest. Upregulation of the cytoskeletal protein, palladin, has been detected in the stromal myofibroblasts surrounding many solid cancers and in expression screens for genes involved in invasion. Using a pancreatic cancer model, we investigated the functional consequence of overexpression of exogenous palladin in normal fibroblasts *in vitro* and its effect on the early stages of tumor invasion.

**Principal Findings:**

Palladin expression in stromal fibroblasts occurs very early in tumorigenesis. *In vivo*, concordant expression of palladin and the myofibroblast marker, alpha smooth muscle actin (α-SMA), occurs early at the dysplastic stages in peri-tumoral stroma and progressively increases in pancreatic tumorigenesis. *In vitro* introduction of exogenous 90 kD palladin into normal human dermal fibroblasts (HDFs) induces activation of stromal fibroblasts into myofibroblasts as marked by induction of α-SMA and vimentin, and through the physical change of cell morphology. Moreover, palladin expression in the fibroblasts enhances cellular migration, invasion through the extracellular matrix, and creation of tunnels through which cancer cells can follow. The fibroblast invasion and creation of tunnels results from the development of invadopodia-like cellular protrusions which express invadopodia proteins and proteolytic enzymes. Palladin expression in fibroblasts is triggered by the co-culture of normal fibroblasts with k-ras-expressing epithelial cells.

**Conclusions:**

Overall, palladin expression can impart myofibroblast properties, in turn promoting the invasive potential of these peri-tumoral cells with invadopodia-driven degradation of extracellular matrix. Palladin expression in fibroblasts can be triggered by k-ras expression in adjacent epithelial cells. This data supports a model whereby palladin-activated fibroblasts facilitate stromal-dependent metastasis and outgrowth of tumorigenic epithelium.

## Introduction

Fibroblasts play a pivotal role in cancer invasion, metastasis, and chemoresistance [1]–[7]. Cancer-associated fibroblasts are myofibroblasts with contractile properties and alpha-smooth muscle actin (α-SMA) staining is a hallmark of these cells [8]. The mechanism by which myofibroblasts enhance tumorigenesis is underscored by three key studies that reveal: 1) cancer-associated fibroblasts chaperone the cancer cells from the primary site into the metastatic niche 2) blocking the activated fibroblasts *before* tumor invasion initiates can prevent cancer; but stopping the myofibroblasts after invasion has started is too late to prevent cancer and 3) therapeutic treatment of pancreatic cancer that reduces the cancer-associated fibroblasts is more effective in prolonging survival than standard chemotherapy that targets only the cancer cells [9]–[11].

The 90 kD isoform of palladin, an embryonic and cytoskeletal protein vital to cell motility, is overexpressed in the cancer-associated fibroblasts of a multitude of tumor types including pancreas, breast, lung, kidney, and ovary but is expressed at lower levels in normal stromal fibroblasts [12]–[15]. Palladin-expressing fibroblasts are also found adjacent to cancer cells in lymph node and liver metastases [12]. Dysregulation of palladin from cultured cells results in aberrant actin organization, dysregulated cell adhesion and motility, and gross disruption of cell morphology [16]–[20]. Not surprisingly, palladin has been detected in expression screens for invasion-specific genes in pancreatic and breast cancer [21], [22].

An interesting association between cancer-associated fibroblasts and palladin in the setting of pancreatic cancer has come to light. We have reported a highly penetrant, rare form of familial pancreatic cancer (Family X) that is caused by a mutation in a highly conserved region of 90 kD palladin. This mutation induces cytoskeletal abnormalities and enhances migration when transfected into cells that normally express minimal amounts of the 90 kD palladin isoform [19]. It was intriguing to find that the palladin protein is overexpressed preferentially and ubiquitously in the stromal compartment of pancreatic cancer rather than the ductal epithelial cells [12], [23].

The fundamental role of palladin in cell motility and the rising awareness that activated fibroblasts can actually partner with cancer cells to promote invasion and metastasis led to these investigations. Herein, using pancreatic cancer as a model, we unravel 1) *when* in neoplastic progression does palladin activate fibroblasts, 2) the *mechanism* underlying the transition of the normal fibroblast into an activated myofibroblast in the setting of cancer and 3) *how* the myofibroblast could aid the cancer cells to escape. In addition, we also explored the effects of an inherited mutated 90 kD palladin in the fibroblasts of a kindred predisposed to pancreatic cancer (Family X or FX). Could a palladin-mutated stromal fibroblast initiate cancer?

## Results

### Palladin expression in tumor-associated fibroblasts occurs early in neoplastic progression and co-localizes with α-SMA in human pancreatic cancer

**Figure 1:**
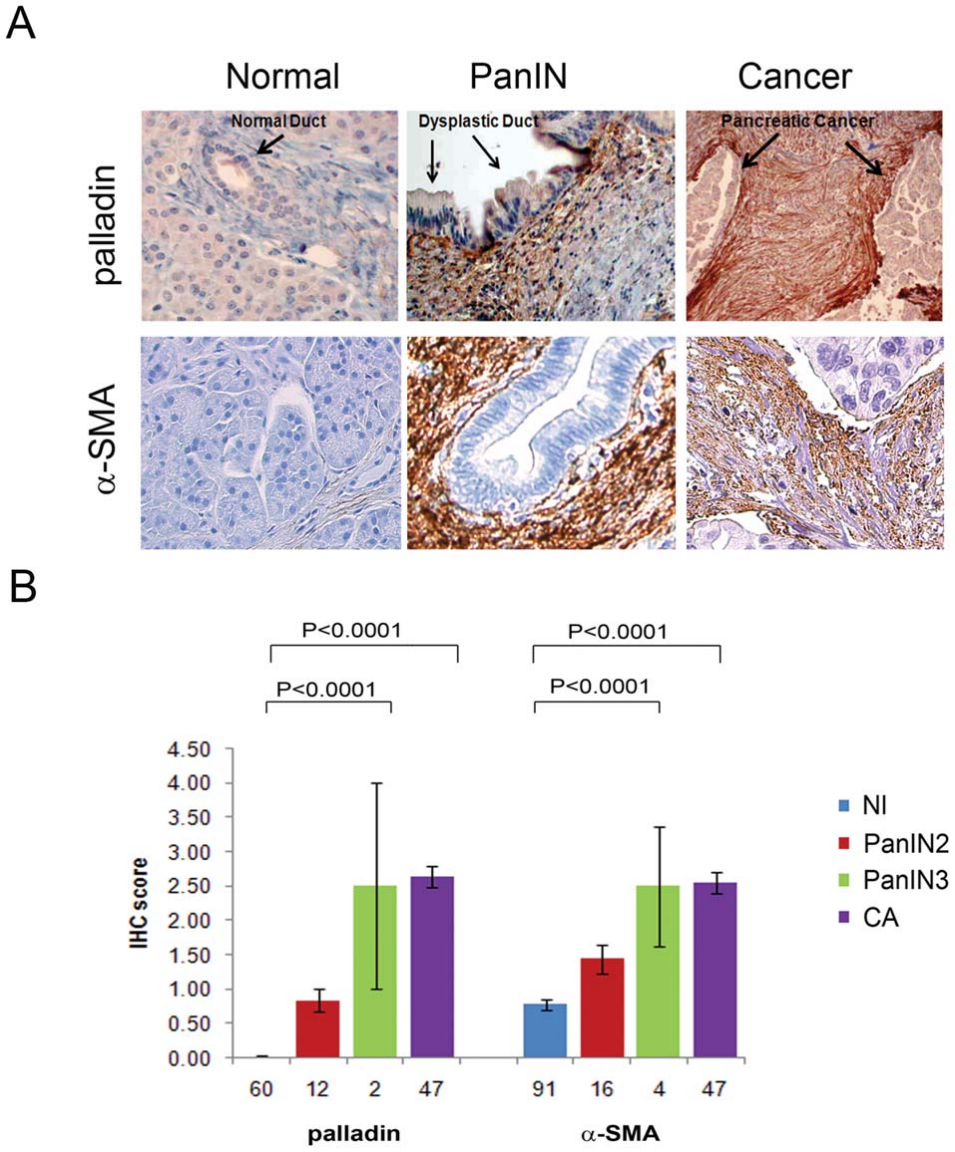
90 kD palladin and α-SMA staining increase with progression of pancreatic tumorigenesis. **A**) Expression of palladin and α-SMA was examined via IHC staining in pancreas specimens. Left: there is little to no staining in normal pancreas. Middle: expression increases in the peri-tumoral fibroblasts of pancreatic dysplasia or PanIN 2. Right: expression is highest and most widespread in the fibroblasts surrounding pancreatic cancer. **B**) Semi-quantitative readings provide IHC scores for tissue microarray staining of normal (NL), low-grade dysplasia (PanIN 2), high-grade dysplasia (PanIN 3) and cancer (CA) by palladin and α-SMA. Staining of palladin or α-SMA was scored as 0 to 4. See also Table S1.

Immunohistochemical (IHC) staining of myofibroblast marker, α-SMA, and 90 kD palladin were performed concomitantly on human tissue microarray blocks containing all histological stages of human pancreatic cancer including precancerous lesions (Figure 1). Normal pancreas lacked 90 kD palladin protein expression except in the lining of the endothelial cells. By contrast, 90 kD palladin expression increased with neoplastic progression in the pre-cancerous dysplastic lesions and the most striking feature was that palladin staining was limited to the fibroblasts immediately adjacent to the dysplastic ductal cells (Figure 1). In pancreatic cancer, diffuse and strong palladin expression was observed throughout the stroma, particularly in the area surrounding the adenocarcinoma cells, in agreement with by previous reports [12], [23]. Expression of α-SMA in the cancer stroma closely paralleled that of palladin as previously reported in renal cell carcinoma [15]. Co-expression of α-SMA and palladin in the fibroblasts surrounding the pre-cancerous lesions suggests that the myofibroblast phenotype is activated early in neoplastic progression and becomes more widespread in cancer.

### Palladin up-regulates α-SMA and activates fibroblasts to become myofibroblasts

**Figure 2:**
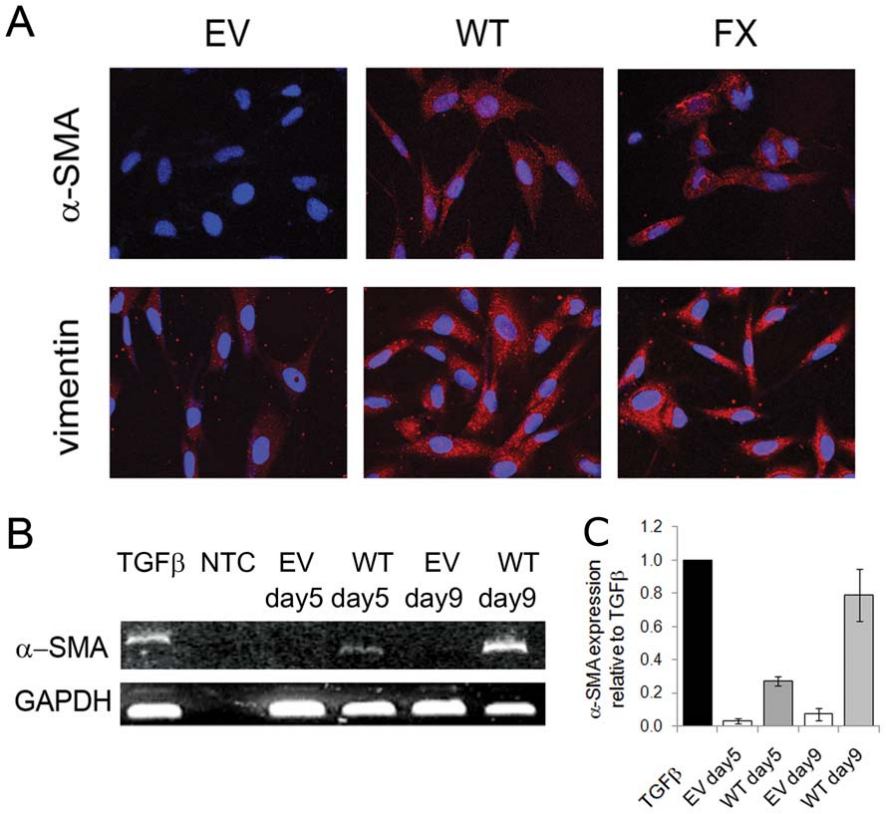
Palladin expression is sufficient to induce normal human dermal fibroblasts (HDF) cells to become myofibroblasts. **A**) HDF cells transfected with 90 kD palladin (WT or FX) or empty vector (EV) were examined for the myofibroblast markers α-SMA and vimentin via IF. Red = α-SMA or vimentin; Blue = DAPI. **B**) HDF cells were treated as above in (**A**) and the expression of α-SMA was analyzed by RT-PCR. RNA was harvested at the number of days indicated post-transfection. **C**) Expression of α-SMA was analyzed by RT-PCR. GAPDH was used as an internal loading control and the relative expression of each sample versus TGF-β1 treated samples were plotted as fold induction ± SD.

Based on the protein expression of palladin and α-SMA in peri-tumoral fibroblasts, we sought to unravel the relationship between these proteins. Palladin is markedly up-regulated during the fibroblast-to-myofibroblast transition following treatment with TGF-β1 [4] as is α-SMA [3]. In vascular smooth muscle cells, palladin expression is required for α-SMA upregulation [24], [25]. We wondered what regulatory effect these two myofibroblast-associated proteins could have upon each other and the myofibroblast phenotype (see data below). Transfection of the 90 kD isoform of wild-type (WT) or mutant P239S (FX) palladin into normal human dermal fibroblasts (HDFs) up-regulates myofibroblast markers, α-SMA and vimentin, and is sufficient to trigger a myofibroblast phenotype following palladin induction. By day 5 post-palladin transfection, α-SMA is significantly elevated (27.4%±2.8%) and even further increased by day 9 (79%±15.7%) (Figure 2). Transfection of palladin also resulted in upregulation of 11 myofibroblast contractile proteins reported by Malstrom et al. [26] (including cofilin, vimentin, profilin, caldesmon, tropomyosin, alpha enolase, beta actin, tubulin, Rho GTP dissociation inhibitor 1, calponin, and peptidyl-prolyl cis-trans isomerase A). This proteomics analysis is discussed in greater detail later.

### Palladin-activated fibroblasts develop a myofibroblast phenotype

**Figure 3:**
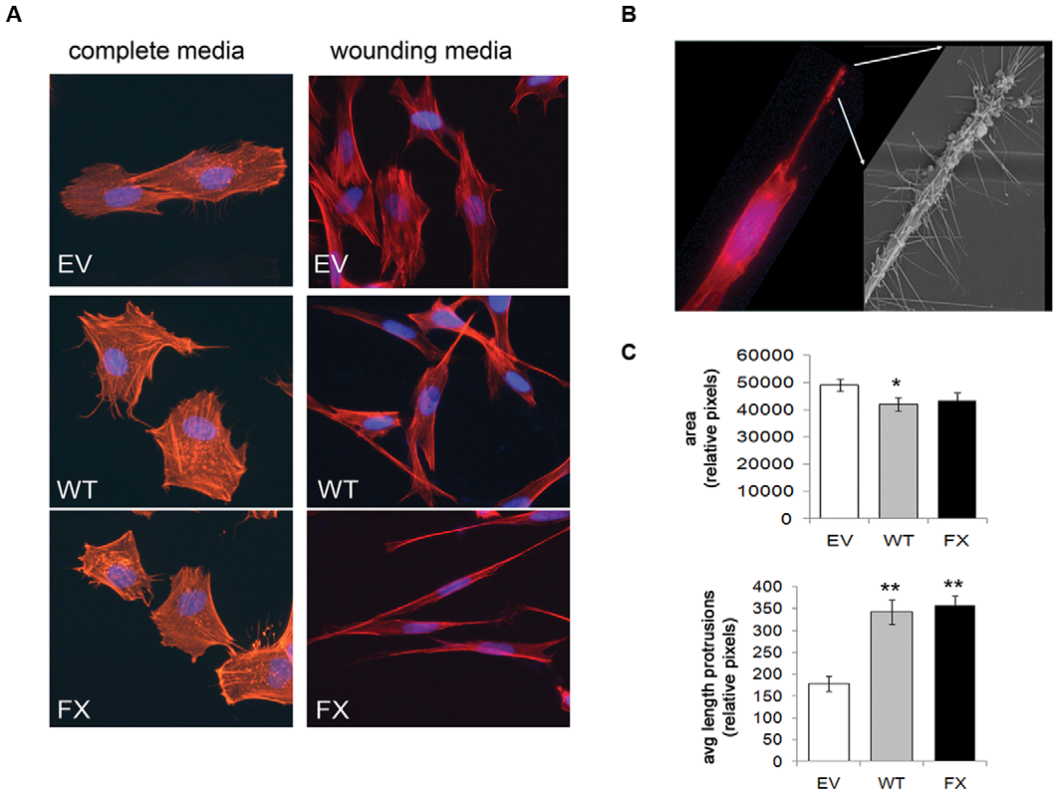
Morphological changes in palladin-expressing fibroblasts with and without exposure to wounding media. **A**) HDF transfected with WT-palladin (WT) or FX-palladin (FX) were compared to empty vector (EV) using IF analysis of phalloidin stained cells grown in complete media or wounding media. **B**) Phalloidin-stained HDF-WT cell protrusion was magnified by electron micrography (*inset*). **C**) HDF were treated as above in (**A**) and cultured in wounding media. Phalloidin stained cells were analyzed by IF. Plots illustrate the relative surface area of cells (*top*) and average length of protrusions (*bottom*). Data are representative of two independent experiments; values are expressed as the mean ± SEM. (t-test, *, p-value<0.05; **, p-value<0.01).

Introduction of 90 kD palladin (WT or FX) into HDF cells (subsequently referred to as HDF-WT or HDF-FX) resulted in alterations to the cellular morphology (Figure 3A). Under normal growth conditions (with complete media), palladin-expressing fibroblasts demonstrated a decrease in cell area, and an increase in thin cellular protrusions with spike-like appendages compared to the parental control cells. Cytoskeletal changes included both lengthening and thickening/bundling of actin stress fibers (Figure 3A). Thin cellular protrusions with spike-like appendages are visible by electron microscopy in the palladin-expressing fibroblasts (Figure 3B). Because many cancers form in an inflammatory setting, and because cancer is considered the wound that doesn't heal [27], we also examined the palladin-expressing fibroblasts after incubation in wounding media (conditioned media from fibroblasts that were wounded with a pipette tip) to determine if further changes were notable (Figure 3A). The morphologic changes were markedly enhanced compared to the empty-vector control fibroblasts (Figure 3C). The cells developed a more fusiform or mesenchymal shape with long thin protrusions compared to the empty-vector control fibroblasts (Figure 3C).

### Palladin-expressing fibroblasts plus a stimulatory trigger enhances invasive and migratory behavior

**Figure 4:**
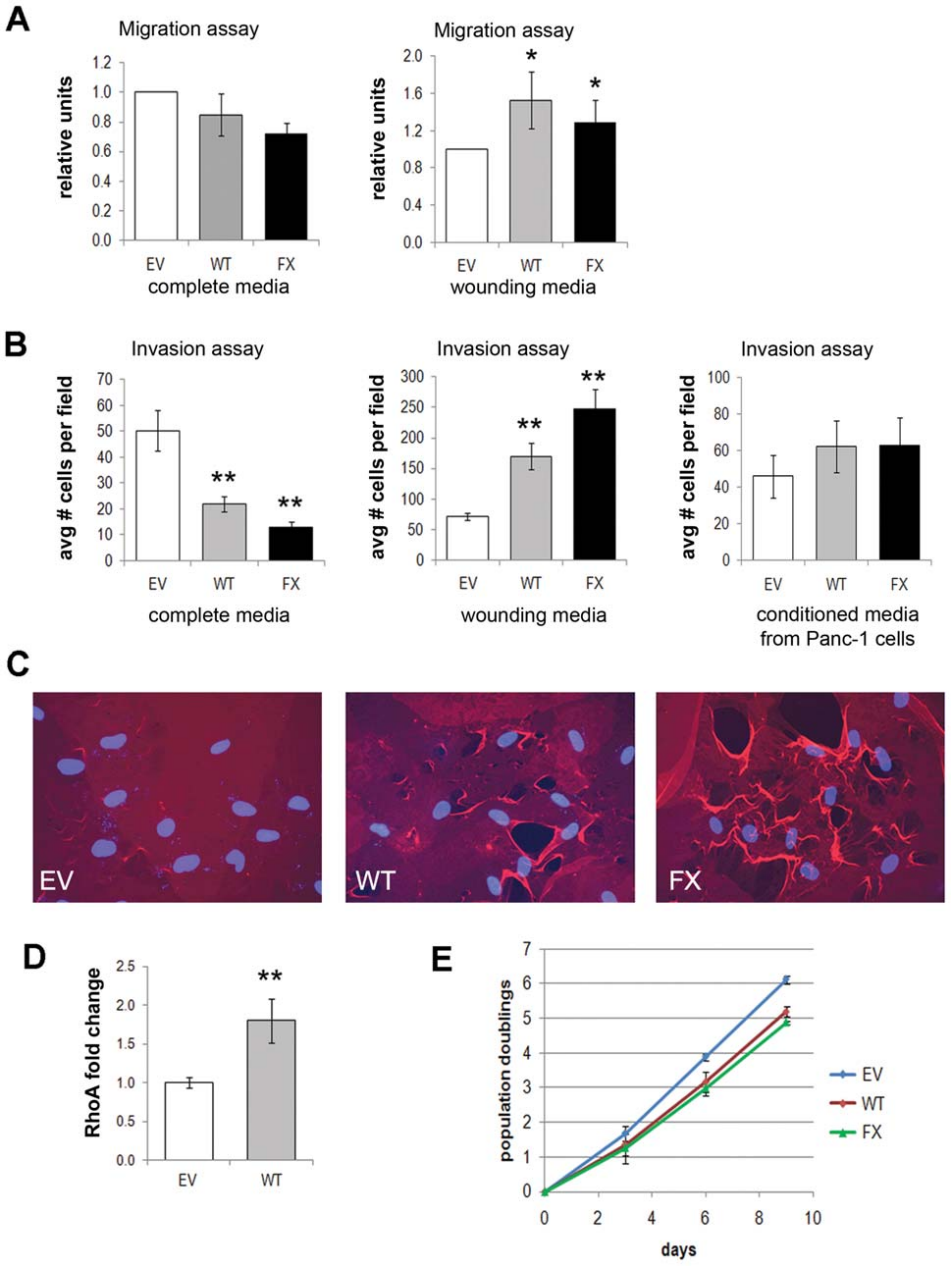
Palladin and a stimulatory trigger enhance HDF cell migration, invasion, and degradation of extracellular matrix. **A**) Migration across a transwell was compared for HDF transfected with empty vector (EV), WT-palladin (WT), FX-palladin (FX) when exposed to normal complete media or wounding media. Data shown is representative average ± SEM of three independent experiments. (t-test, *, p-value<0.05) **B**) Invasion across a matrigel-covered transwell was compared for HDF cells treated as above in (**A**) when exposed to complete media, wounding media, or conditioned media from Panc-1 cells. Data shown is representative average ± SEM of three independent experiments. (t-test, **, p-value<0.01) **C**) HDF cells were transfected as above in (**A**) and plated onto coverslips coated with Texas Red-labeled gelatin. Representative images taken via IF are shown. DAPI (blue) was used to visualize nuclei. **D**) Protein lysates from HDF cells transfected as above in (**A**) were tested for RhoA activity. Each sample was run in triplicate in two independent experiments. Error bars indicate SD. (t-test, **, p-value<0.01) **E**) Proliferation assay of HDF cells transfected as above in (**A**). Data indicates mean ± SD for three independent wells.

We next asked whether 90 kD palladin expression would affect the migration and/or invasive capacity of fibroblasts. Migration was tested using a transwell system where the cells can traverse directly through pores from an upper chamber to a lower chamber. Invasion was tested using a Matrigel-coated transwell. Assay results were compared for incubation with complete media, wounding media (conditioned media collected from confluent HDF cells manually wounded with a pipet tip), or conditioned media from Panc-1 epithelial cells. Previous studies have shown that exposure of cells to wounding media, increases the migration rate of epithelial cells [28]. In agreement with recent studies showing anti-migratory effects of palladin expression in breast cancer cells [29], in the absence of a wounding signal, palladin-activated fibroblasts did not have enhanced migration or invasion. However, a wounding signal dramatically increased the migration and the invasive capacity of palladin-activated fibroblasts (WT and FX) compared to empty vector control (HDF-EV) (Figures 4A & 4B). While epidermal growth factor (EGF) had a similar result on the cells as wounded media (data not shown), incubation with conditioned media from pancreatic cancer cells (Panc-1) did not enhance the invasiveness of palladin-expressing cells (Figure 4B).

To verify whether palladin-expressing cells were capable of degrading extracellular matrix, HDF-EV, HDF-WT, and HDF-FX were grown on coverslips coated with fluorescently-labeled gelatin. In the presence of wounding media, gelatin bundling was observed and the gelatin surface was destroyed by the palladin-expressing HDF-WT or HDF-FX cells, but not the normal parental HDF-EV (Figure 4C). These destructive changes are much more dramatic and widespread than would normally be expected with invadopodia alone. We wondered if the palladin-induced myofibroblasts had enhanced contractility that could cause a “ripping” of the matrix. To answer this, we investigated whether RhoA, a protein involved in cytoskeletal movement and remodeling [30], was activated in the palladin-expressing cells and found that RhoA levels were increased nearly 2-fold (Figure 4D). This suggests that exogenous palladin expression promotes the ability of myofibroblasts to both invade and destroy extracellular matrix (Figure 4C). The invasive effects of wounding media and EGF on palladin-expressing fibroblasts were not due to a difference in proliferation rates (Figure 4E).

**Figure 5:**
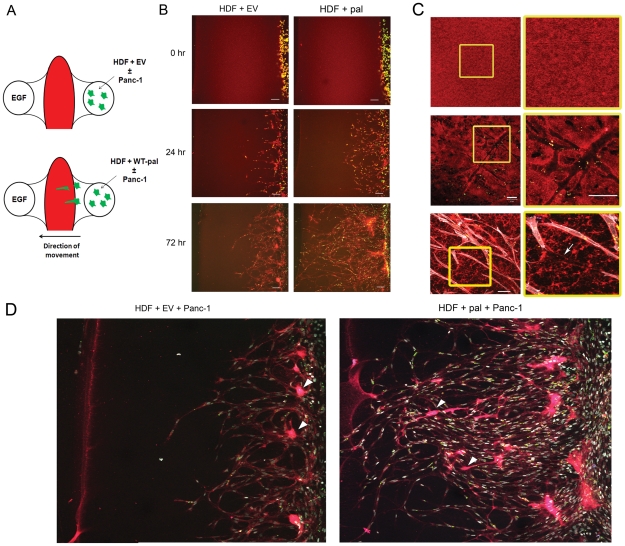
Palladin-activated fibroblasts lead Panc-1 cells through the extracellular matrix. **A**) Schematic of 3-D invasion assay showing Texas Red labeled gelatin/matrigel mixture in the channel with 5 ng/ml EGF as an attractant in the left port and cells (HDF ± palladin labeled with QTracker 585 with or without Panc-1 labeled with QTracker655) seeded into the right port. Cell movement was evaluated as cells traversed the channel (right to left directional movement) using confocal microscopy. **B**) Palladin-activated fibroblasts (green) invade further into the red matrigel channel at all time points tested (24 and 72 hours) *(Right panels)* compared to HDF with EV *(Left panels)*. Representative images taken with confocal microscopy are shown. Bars indicate 100 µm. **C**) The yellow boxed region is shown at higher magnification to the right. *top*, Red matrigel is uniform in the empty channel prior to invasion. *middle*, Palladin-activated fibroblasts (green fluorescence emission) created black tunnels (devoid of Texas Red signal; see arrow) within the matrix. *Bottom*, Fibroblasts (white phalloidin stain) can move single file through the tunnels. Shown are representative images taken with confocal microscopy. Bars indicate 50 µm. **D**) Panc-1 cells (pink; see arrowheads) follow palladin-activated fibroblasts (white) through the channel while they do not follow the HDF-EV. Shown are representative images taken with confocal microscopy 72 hours after co-culture. Channels were fixed and stained with DAPI.

Previous investigators have suggested that tumor-associated fibroblasts may lead cancer cells through the extracellular matrix [31]. We queried whether the palladin-activated fibroblasts could fit that paradigm. To examine this hypothesis, we performed 3D invasion assays using slides developed by Bellbrook Labs (Figure 5A). Two circular wells were connected by a central straight channel of 1 mm×1.8 mm. The channel was filled diluted fluorescent matrigel; EGF was added to the well on one side of the channel as an attractant and HDF or palladin-activated HDF were loaded into the opposite well. Cell invasion into the matrigel-filled channel was assessed at 12 hour intervals as fibroblasts moved toward the attractant. In agreement with our transwell invasion assays, palladin-activated fibroblasts invaded the channels more quickly and migrated across the channel in significantly greater numbers than control fibroblasts (Figure 5B). Even with extended time, none of the HDF cells without palladin were able to cross the channel. In contrast, the palladin-activated fibroblasts completely crossed the 1 mm channel by 72 hours, creating tunnels that were used by other activated fibroblasts that followed behind. The black tunnels created by the palladin-activated fibroblasts are quite clearly seen in the fluorescent-red channel of the 3D invasion chamber (Figure 5C). In addition to the extensive tunneling created by the palladin-activated fibroblasts toward the attractant, the palladin-containing cells appeared to have better directional movement towards the EGF attractant than the control cells (Figure 5B); the latter frequently circled back to the home well where they started.

Panc-1 cells were added one day after the fibroblasts to evaluate whether these epithelial cells would make use of the tunnels previously created by the invading fibroblasts. Indeed, we were able to identify Panc-1 cells following the palladin-activated fibroblast cells through the tunnels (Figure 5D). Palladin-activated fibroblasts had a direct influence on the behavior and appearance of the Panc-1 pancreatic cancer epithelial cells. After co-culture of Panc-1 and palladin-activated fibroblasts cells in the home well of the 3D-invasion slides, the Panc-1 cancer cells migrated a much further distance– some completely crossing the channel– while the control palladin-free co-cultures revealed the Panc-1 cells barely leaving the home well. In addition, in the presence of palladin-activated fibroblasts, the Panc-1 cells developed an elongated appearance (Figure 5D).

### Palladin promotes invasion through the development of invadopodia filled with matrix-destroying enzymes

**Figure 6:**
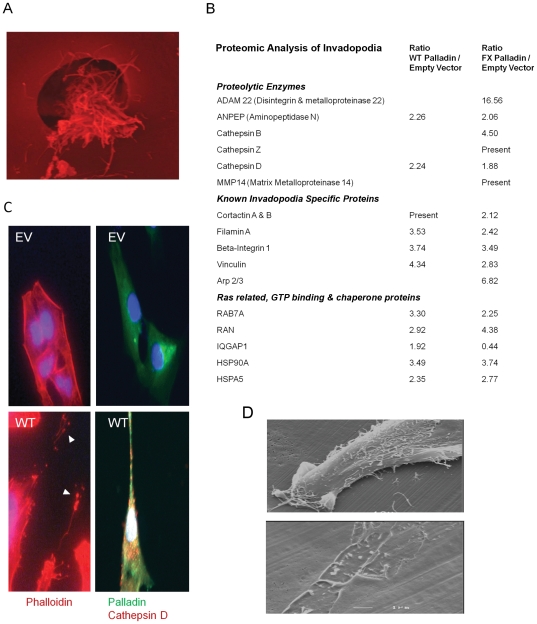
Palladin-activated fibroblasts use invadopodia to degrade extracellular matrix. **A**) The underside of a 3 µm transwell shows podosomes/invadopodia from an HDF-WT fibroblast that has invaded through the matrigel via IF. The “feet” were severed with a razor for proteomic analysis. **B**) HDF cells transfected with GFP-empty vector (EV) or GFP-wildtype palladin (WT) were examined with phalloidin staining via IF. Palladin-expressing fibroblasts have linear protrusions (*arrowheads*) filled with proteases and lysozymes such as Cathepsin D. Shown are representative images. **C**) Proteomic analysis of the “feet” reveals overexpression of proteolytic enzymes, Rho activation proteins, and verifies the presence of proteins previously identified in invadopodia. Shown are protein ratios from fibroblasts with WT or FX palladin relative to empty vector. See also Table S2. **D**) HDF cells transfected with wildtype palladin were grown on matrigel covered coverslips. Erosive degraded tracks were detected via electron microscopy. Bar indicates 1 µm.

To unravel the mechanism underlying the invasive capability of the palladin-expressing fibroblasts, we examined the “feet” of the palladin-activated fibroblasts, which had become more prominent with the transition into myofibroblasts (Figure 3B). HDF, transfected with and without palladin, were plated on Matrigel-coated transwells. Although the pore size of the transwell was too small for an entire cell to go through, it could accommodate the podosomes, which had to first penetrate the Matrigel layer (Figure 6A). The invadopodia/podosomes that invaded through the matrix-covered pores were severed from the cell body with a razor. Labeled quantitative proteomic analysis was used to compare the proteins in the “feet” from palladin-expressing fibroblasts relative to the “feet” from the normal parental fibroblasts. Proteomic analysis revealed that over 200 proteins were dysregulated by ≥1.5 fold in the “feet” of palladin-expressing fibroblasts relative to those of labeled control cells. These dysregulated proteins including invadopodia proteins [30], [32], [33], ras related and GTP binding proteins, and proteolytic enzymes, (selected proteins, see Figure 6B; for complete list, see Table S2). We were particularly interested in the invadopodia proteins, including cortactin, filamin A, beta-integrin 1, vinculin, profilin, alpha-actinin, and cofilin [32], [33]. Nearly half of the up-regulated proteins identified in our proteomic analysis were recently reported as novel invadopodia components [34].

Quantitative proteomic analysis revealed up-regulation of the palladin-binding protein, alpha-actinin which is another invadopodia-related protein that has been associated with poor prognosis in ovarian and pancreas [35], [36]. The marked increase of alpha-actinin in the stroma of the pancreatic cancer and pre-cancer was validated by IHC (Figure S1). Validation of the increased expression of selected invadopodia proteins –cofilin, cortactin, and profilin–detected by the proteomics analysis was performed by immunofluorescence and/or Western blot analysis (Figure S2).

In addition to invadopodia proteins, proteolytic enzymes were also up-regulated in the “feet” of palladin-expressing fibroblasts including ADAM22, aminopeptidases, and cathepsin D and B (Figure 6C). Immunofluorescent staining of the cathepsin D, confirmed that the protein is selectively overexpressed in the HDF-FX and WT cells, but not in the parental fibroblasts with empty vector (Figure 6C). Interestingly, cathepsin D has been shown to stimulate fibroblast invasion and outgrowth and is reportedly involved in paracrine interactions between tumor epithelium and fibroblasts [37]. In addition to the expression of proteolytic and matrix-remodeling proteins, destruction of matrigel by the palladin-expressing HDF-WT cells is evident by electron microscopy (Figure 6D).

### Palladin is up-regulated in fibroblasts by the adjacent k-ras activated epithelial cells

**Figure 7:**
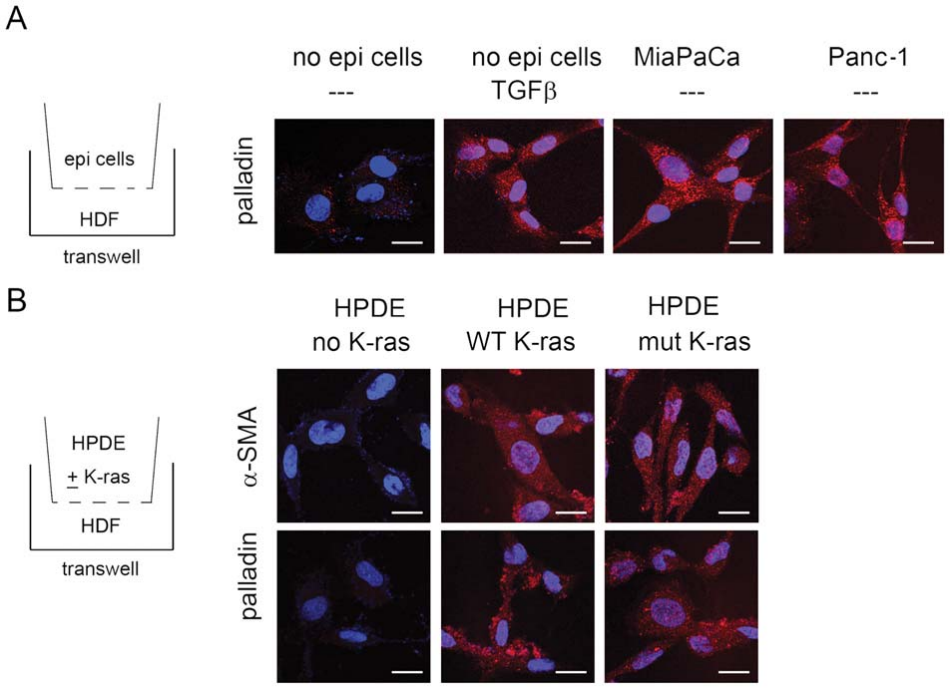
Palladin is up-regulated in normal fibroblasts co-cultured with pancreatic cancer or normal epithelial cells containing K-ras. **A**) HDF cells were examined for palladin expression via IF following co-culture with pancreatic cancer cell lines, MiaPaCa or Panc-1, for 7 days. TGFβ1 is shown as a positive control for up-regulating palladin in normal fibroblasts, no epithelial cells is the negative control. Scale bars indicate 20 µm. **B**) HDF cells were examined for α-SMA or palladin expression via IF following co-culture with normal pancreatic duct epithelial cells (HPDE) that were mock transfected or transfected with wild-type or mutant K-ras. Scale bars indicate 20 µm.

Palladin is generally not mutated in sporadic pancreatic cancer (data not shown) or in familial pancreatic cancers, other than Family X [38]–[41]. Yet expression of 90 kD palladin has been reported in the fibroblasts that surrounded pancreatic dysplastic or cancerous ductal cells in 96% of pancreatic cancers, while the fibroblasts that surround normal ductal cells do not [12], [23] (Figure 1). We hypothesized that paracrine signaling between the cancerous epithelial cells and fibroblasts was likely responsible for the up-regulation of palladin expression in the fibroblasts, eventually turning them into tumor-associated myofibroblasts. To test this hypothesis, normal human fibroblasts were co-cultured in a transwell with immortalized normal pancreatic ductal cells (HPDE cells) and with pancreatic ductal cancer cell lines which have k-ras mutations (MiaPaCa and Panc-1). By day 5 of co-culture, palladin was up-regulated in the fibroblasts adjacent to both of the pancreatic cancer ductal cell lines (Figure 7A). By comparison, normal fibroblasts that were adjacent to normal pancreatic ductal cells did not express palladin (Figure 7B).

Next we wondered what the signal was from the cancer cells that would up-regulate palladin in adjacent fibroblasts? In light of our data showing the earliest time course for the up-regulation of palladin in the peri-tumoral fibroblasts occurs at low-grade dysplasia (known as PanIN 2 in the pancreas), we hypothesized that palladin regulation must occur relatively early in tumorigenesis. K-ras mutations are ubiquitous in pancreatic cancer: present in both the cancerous and pre-cancerous ductal cells [42]. Because palladin over-expression is noted in the myofibroblasts immediately adjacent to the pre-cancerous ductal cells (Figure 1), we speculated whether k-ras activation alone in ductal cells was sufficient to cause up-regulation of 90 kD palladin in neighboring normal fibroblasts. A similar co-culture experiment using the transwells was devised with the normal fibroblasts in the lower chamber; the upper chamber contained normal pancreatic ductal epithelial cells (HPDE) transfected with wild-type k-ras, mutated k-ras, or an empty-vector. Expression of either constituently activated wild-type *or* mutated k-ras in HPDE cells was sufficient to cause the up-regulation of palladin and α-SMA in the adjacent normal fibroblasts (Figure 7B). Neither palladin nor α-SMA expression were increased upon co-culture with parental pancreatic ductal cells (HPDE) transfected with an empty vector.

### Upregulation of α-SMA and palladin in fibroblasts by the adjacent cancer cells is prevented by silencing of 90 kD palladin

**Figure 8:**
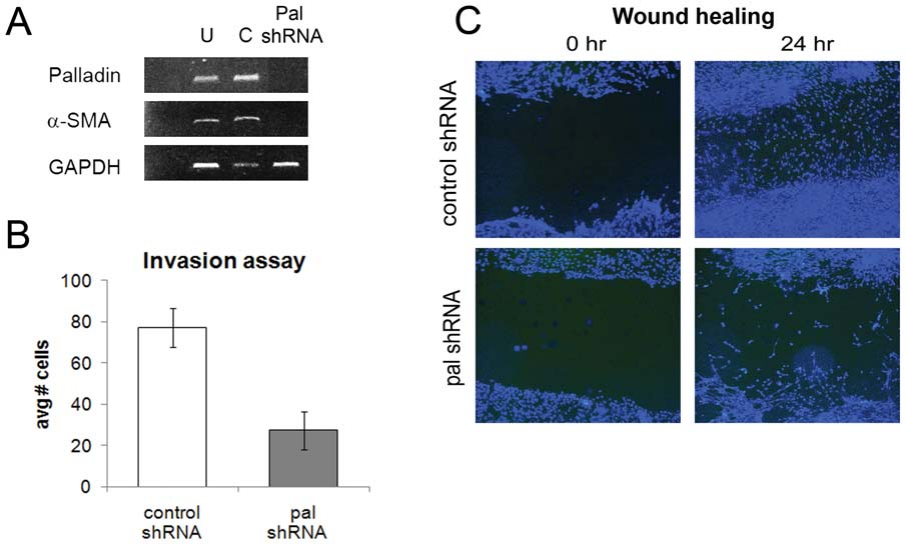
Silencing of Palladin abrogates the myofibroblast phenotype and function. **A**) HDF cells were stably transfected with control shRNA (C) or shRNA against 90 kD palladin (Pal shRNA). α-SMA expression following co-culture with Panc-1 cells was analyzed via RT-PCR. Analysis of untreated HDF (U) is shown for comparison. The first lane is a non-template negative control; GAPDH is shown as an internal loading control. Shown is data from one representative shRNA stable clone (of three independent shRNA constructs tested). **B**) Invasion across a matrigel coated transwell was compared for HDF transfected as in (**A**). Shown is the mean ±SD. Shown is data from one representative shRNA stable clone (of three shRNA constructs tested). **C**) HDF cells as in (**A**) were grown to confluence on coverslips and wounded with scratch test. Migration was assessed via IF 24 hours after wounding. At least 3 observations for each condition were analyzed. Blue = DAPI. Shown is data from one representative shRNA stable clone (of three shRNA constructs tested).

To determine whether the ras-induced transformation of fibroblasts into myofibroblasts relied on a palladin dependent pathway, we utilized shRNA to generate stable clones of HDF with palladin knockdown and then measured palladin and α-SMA production after 9 days of co-culture of the normal fibroblasts with the pancreatic cancer cell line, Panc-1. We failed to detect α-SMA expression by RT-PCR following co-culture of HDF cells stably transfected with any of three distinct shRNA constructs targeting 90 kD palladin (Figure 8A; data not shown). However, upregulation of α-SMA was observed upon co-culture with Panc-1 cells if HDF cells were transfected with a control shRNA plasmid or in untreated HDF cells. There was also a functional consequence to silencing palladin in fibroblasts, as demonstrated by migration and invasion assays (Figures 8B & 8C). Palladin silencing diminished the functions previously associated with the myofibroblast phenotype. Together these findings indicate that ductal cells containing activated k-ras are sufficient to up-regulate palladin expression in adjacent fibroblasts through paracrine signaling. This in turn causes the fibroblast to transform into a myofibroblast. Complete silencing of palladin abrogates the process.

### Mutated palladin (FX) confers some differences in protein expression and behavior in fibroblasts compared to wild-type palladin (WT)

For several reasons, we included in these experiments the study of the P239S mutation in a highly conserved region of 90 kD palladin that causes a rare form of autosomal dominant familial pancreatic cancer. How could dysregulation of a cytoskeletal protein in peri-tumoral fibroblasts predispose to such a lethal, highly penetrant cancer? In these studies, we found that the FX palladin expressing fibroblasts were significantly more invasive than fibroblasts containing wild-type palladin. In addition, quantitative proteome analysis detected expression of 88 proteins that were unique to the HDF-FX (Table S2) and not detected in HDF-WT. IHC of Family X pancreas reveals that low levels of palladin are overexpressed in some of the normal appearing fibroblasts adjacent to normal ducts, while this is not the case in normal pancreas of donor controls (unpublished observations). Of note, the Family X cancers all had k-ras mutations (data not shown), as is seen in the sporadic form of the disease. Collectively, these findings suggest that the FX palladin mutation confers a distinct advantage from the myofibroblast side of the neoplastic equation but that the fundamental genetic alterations, such as k-ras mutation, are still required from the ductal cells for neoplastic progression.

## Discussion

Who would have thought that when looking at a pathology slide of pancreatic or breast cancer, the vast field of fibroblasts surrounding the cancer cells was anything but a scarring response to the damaging cancer cells? The importance of stroma was suggested decades ago, but only recently have biologists delved deep into the role that cancer-associated fibroblasts play in all stages of cancer including initiation, progression and metastasis [1]–[4], [6], [7], [43]–[45].

Physically, how can fibroblasts assist a cancer cell? Elegant studies by Gaggioli have demonstrated that the myofibroblasts create tunnels through the matrix– clearing a path that allows the cancer cells to follow behind, much as cars could follow a snowplow [31], [46]. Through the work presented here, the mechanism underlying myofibroblast-led tumor invasion becomes clearer. Palladin activates fibroblasts inducing them to become myofibroblasts. These palladin-expressing cells develop a different phenotype and proteomic profile compared to normal fibroblasts. The cells develop streamlined body shapes and innumerable invadopodia filled with destructive enzymes. With a wounding signal, the activated fibroblasts become invasive; their increased contractility allows them to literally rip through tissue while at the same time, the developed invadopodia destroy the matrix. While we have not identified which factor(s) in the wounding media contributes to the enhanced migration and invasive capacities observed here, this would be an area for future investigation.

From our invasion assays, we observed that palladin-activated fibroblasts could invade through matrigel a distance approximately 600 µm within a 24 hour period (Figure 5). If the average human pancreas is 15 cm in length, this data would suggest that these activated cells could potentially migrate the entire length of the pancreas within 250 days, and could travel to the lymphatic or blood vessels in significantly less time. These tunnels could then also be utilized by tumorigenic epithelium or other cell types. Interestingly, without a wounding signal, the myofibroblasts are *less* motile than a normal fibroblast. Recent studies in mice have demonstrated that PDAC induction is dependent upon both the k-ras oncogene and a STAT3-dependent inflammatory component, such as chronic pancreatitis [47], [48]. These collective observations might allude to why a cancer could remain dormant versus invasive over time: without a trigger/signal propelling forth the activated and invasive fibroblasts, the cancer cells are significantly less invasive. Conceptually, such a trigger-signal could be a growth factor or chemokines, such as EGF, or wounding media, as we show in our studies.

### Activation of palladin early in tumorigenesis

Using pancreatic cancer as a model, we determined that tumor-associated fibroblasts express palladin very early in tumorigenesis, when ductal cells have low-grade dysplasia, as demonstrated previously in a mouse model of pancreatic cancer [12]. Moreover, the number of palladin-expressing fibroblasts expands with neoplastic progression to involve all of the stromal fibroblasts by the time that cancer forms. We demonstrate that k-ras activation or mutation in the epithelial cells is sufficient to induce up-regulation of palladin in the adjacent normal fibroblasts and transform them into myofibroblasts within 5 days. Studies by Logsdon have shown that the degree of k-ras expression in an epithelial cell positively correlates with the level of dysplasia of that epithelial cell [49]. One could hypothesize that as k-ras signaling is amplified with neoplastic progression of the epithelial cell; palladin expression is augmented in fibroblasts to reach an exuberant level by the time that cancer develops. One drives the other. Our IHC studies demonstrating that palladin expression in the stroma increases with advancing tumorigenesis would certainly fit with this hypothesis. Future studies could help unravel the biochemistry that underlies the ras/palladin relationship.

### Initiation of cancer

Can a fibroblast initiate a cancer? Some data suggests this is possible. Cancer-associated fibroblasts that are mixed with non-tumorigenic prostate epithelial cells can induce tumorigenesis in mouse models [50]. Carcinogen-treated mammary gland stroma, when mixed with unexposed, normal mammary epithelial cells, results in adenocarcinomas in mice [51]. Endometrial stromal cells mutated with APC acquire a myofibroblast phenotype and the stromal cells alone are sufficient to induce endometrial cancer in an engineered mouse model [52]. Conversely, “good” fibroblasts can help prevent tumorigenesis in mouse mammary tumor and liver cancer models [51], [53]; cancer cells that are placed in a normal embryonic microenvironment can be reprogrammed to behave normally [54]. As the tumor-modulating role of stromal cells has been uncovered, investigators have looked for mutational defects in the cancer-associated fibroblasts to account for fibroblast cancer-promoting behavior [55]. Our data suggests at least one simple process to change the behavior of a stromal fibroblast: the paracrine signaling between an epithelial cell and its adjacent fibroblast. Activation of k-ras in an epithelial cell is sufficient to transform a fibroblast into a myofibroblast through palladin expression. One could posit that a *mutation* of k-ras in an epithelial cell would insure that k-ras is in an activated state long-term, providing a continuous signal to the adjacent fibroblast. However, in order to initiate invasion, it appears that the equation requires an activated palladin-expressing fibroblast plus injury, as discussed above. Perhaps this could explain why chronic injury in an organ, such as inflammation, leads to an increased risk of cancer—in such a setting, activated fibroblasts could more easily become deadly partners of cancer cells because the injury is ever-present.

What can Family X, who inherited a mutation in the highly conserved region of 90 kD palladin, tell us about this paradigm of cancer initiation? It appears that the palladin mutation confers a super-invasive behavior of the fibroblasts, but a k-ras mutation is still required. This might explain why family members don't acquire cancer in childhood but rather in mid-adulthood at ages 30–50. It may take decades of time to acquire k-ras mutations in the pancreas and to generate an injury signal such as could be caused by smoking, excess alcohol, or fatty oxidation. The susceptibility of the family members to pancreatic cancer thus would require all 3 elements for pancreatic cancer: k-ras mutation, injury, and palladin-activated fibroblasts; but in the case of Family X, it takes less to initiate or promote a cancer as they already possess one of the required elements: the easily primed, palladin-mutated fibroblasts. In this setting, it seems unlikely that any dysplasia could remain dormant. Without the “good” fibroblast for protection, any spark could ignite the cancer. Future studies could help determine whether an activated fibroblast can confer mutational susceptibility to an adjacent epithelial cell.

The tumor stroma is an ominous partner of the tumor cells: a field of activated myofibroblasts enhances invasiveness and metastases of the surrounded cancer cells. In this study, we demonstrate that introduction of wild-type or mutated (FX) palladin in a normal fibroblast cell line is sufficient to transform the cell into myofibroblasts as evidenced by up-regulation of α-SMA and vimentin. Palladin alters the cell shape into spindle-shaped cells with numerous invadopodia, and alters cell function by enhancing the capacity for migration and invasion. In pancreatic cancer, palladin expression occurs early in neoplastic progression and expands from peri-ductal fibroblasts adjacent to dysplasia to ubiquitous expression throughout the cancer stroma. Fibroblast expression of palladin is induced by k-ras activation in the adjacent ductal cells and the transition of the fibroblast to myofibroblast is palladin-dependent. While palladin expression is sufficient and necessary to induce phenotypic myofibroblast changes, a wounding signal is required to launch the primed cell into an invasive, leading partner for cancer cells. Thus, the large number of myofibroblasts that make up what appears to be static “scar tissue” in some cancers may be, on the contrary, in an active state of movement that extends beyond the organ of origin. Palladin appears to play a central role in the arousal of the fibroblasts in tumorigenesis.

## Methods

### Specimens

Pancreatic tissue specimens were collected in accordance with approved Human Subject's guidelines at the University of Washington and Virginia Mason Hospital in Seattle, and the Cleveland Clinic Foundation in Cleveland. All of the specimens were obtained from surgical resections intra-operatively, immediately processed for paraffin embedding.

### Cell culture and SILAC labeling

Human dermal fibroblasts (HDF), Panc-1 and MiaPaCa cells were maintained in complete DMEM supplemented with 10% fetal bovine serum and 0.01% penecillin-streptomycin in a humidified incubator at 37°C with 5% CO_2_. The normal human pancreatic ductal epithelial cell line (HPDE) was obtained from Dr. Ming-Sound Tsao (University of Toronto, Ontario, Canada). WT and FX palladin (90 kD isoform) constructs were described [19]. Wounding conditioned media was prepared by manually wounding confluent HDF cells and incubating with fresh complete media for 16–18 hours. HDF cells were grown in modified DMEM with ^13^C_6_-lysine and ^13^C_6_-arginine (Thermo Scientific) for SILAC labeling. Cells were transfected as previously described [56]. 24 hours post-transfection, cells were flow sorted for GFP signal on a Cytopeia Influx Flow Cytometer or a BD FACS Aria II. Brightfield images were taken using the 10× objective on a Leica DMLB microscope equipped with a Spot Insight camera using Spot Advanced software.

### Co-culture and palladin knockdown

HDFs were grown in 6 well dishes and transwells (Corning) containing HPDE, Panc-1, or MiaPaCa cells were placed atop HDF cells (day 1). Cells were fed every 2–3 days with complete DMEM and passaged as needed. shRNA plasmids were purchased from Qiagen and transfected into competent JM109 cells (Promega). Transformants were selected with ampicillin and DNA isolated using the Qiagen Endo-Free Plasmid Kit as per manufacturer's instructions. DNA was digested with ScaI (New England Biolabs) overnight at 37°C and cleaned up using the MinElute Clean Up Kit (Qiagen) as per manufacturer's instructions. HDF cells were transfected with linearized ScaI plasmids using the Attractene Reagent (Qiagen). Cells were selected with 10 µg/ml puromycin (Invitrogen) for one week; media was changed every 2–3 days. Stable transfectants were then selected with 100 µg/ml puromycin treatment for 6 weeks with media change every 2–3 days.

### RT-PCR

Total RNA was isolated from cell pellets using the RNeasy Mini kit (Qiagen) as per manufacturer's instructions. 50 ng RNA was used for first strand cDNA synthesis using the SuperScript III First Strand Synthesis System (Invitrogen). 2 ng of cDNA was used for RT-PCR using the FastStart Taq Kit (Roche). Primer sequences and conditions were as described for α-SMA [57]. Palladin was amplified using the following primers: forward, ctgcccaagggtgtcac; reverse, ctttggctttggatttccag. Gels were analyzed using ImageJ to determine relative band intensities.

### Tissue microarray construction

Tissue microarrays were constructed from representative pathologic or normal tissues from paraffin-embedded formalin or Hollande's-fixed samples. Histology of cores was independently verified by pathologists (MPB and JC). For palladin, sections included 47 different cases of sporadic pancreatic ductal adenocarcinoma 20 PanINs, and 91 different cases of normal pancreas (including sections from normal, pancreatitis, and normal sections adjacent to adenocarcinoma). Triplicate 1.5 mm diameter cores of each tissue type were embedded into a systematic grid using a tissue arrayer (Beecher Instruments) as previously described [58].

### IHC

Immunohistochemical staining for smooth muscle actin was performed on 4 µm paraffin-fixed sections with the Benchmark XT, an automated immunostainer from Ventana Medical Systems (VMS), Tucson, AZ, USA. Briefly, the slides were processed for antigen retrieval using microwave heating in a citrate buffer, followed by primary antibody incubation using the 1E6 monoclonal palladin antibody [19] at a titer of 1∶5000 or a mouse monoclonal antibody for smooth muscle actin (Dako) at a dilution of 1∶50. The specific protein-antibody complexes were located using a biotin/streptavidin-HRP/(DAB) detection kit (iView DAB Detection). Scoring guidelines are outlined in Table S1.

### Fluorescence microscopy

For cell morphology experiments, cells were grown on coverslips and stained with Texas Red phalloidin (Invitrogen) diluted 1∶1000. For immunofluorescence, HDF cells were grown on coverslips, fixed with4% paraformaldehyde, and permeabolized briefly with 0.2% Triton X-100. Antibodies against the following proteins were used at the indicated dilutions: Texas Red phalloidin (Invitrogen) 1∶1000; α-SMA rabbit 1A4 (Abcam) 1∶400; palladin (Proteintech) 1∶1000; palladin (Novus Biologicals) 1∶1000; cortactin (Abcam) 1∶500; nestin (Millipore) 1∶200;profilin (BD Biosciences) 1∶400; cofilin (Sigma) 1∶500 AlexaFluor 488 chicken anti-rabbit IgG (Invitrogen) 1∶1000 or Rhodamine Red IgG (Jackson ImmunoResearch) 1∶100. Coverslips were mounted onto glass slides using Prolong Gold+DAPI (Invitrogen) and sealed with nail polish. Images were taken with 63× or 100× objectives on a Leica DMLB microscope equipped with a Diagnostic Instruments Color Mosaic camera and software and analyzed using ImageJ (NIH) or using the 40× or 63× objective and sequential scans of the 405 nm, 488 nm, and 543 nm lasers as appropriate on a Zeiss LSM 510 Meta confocal microscope at the Keck Center for Microscopy at the University of Washington.

### Migration and Invasion assays

The lower sides of the transwell inserts (Corning; 8 µm pores) were coated with 100 µg/ml fibronectin overnight at 37°C. Invasion assays were performed using a modified migration assay [59]; for invasion assays, the upper chambers of the transwells were coated with diluted matrigel (BD Biosciences). For both migration and invasion assays, 4×10^4^ cells were plated in the upper chamber in serum-free media atop wounded HDF in the lower chamber and incubated for 20 hours. Invasion assays were also performed using conditioned media from Panc-1 cells in the lower chamber. For migration assays, cells in the upper chamber were removed and inserts were fixed in ice cold methanol. Transwells stained with crystal violet, and absorbance was measured at 590 nm. For invasion assays, transwells were fixed in ethanol, and mounted onto slides with Prolong Gold plus DAPI (Invitrogen). The number of migrating cells was counted per field and results were calculated as number of cells relative to empty vector control. Each sample was run in triplicate and in multiple experiments. Shown are representative images; graphs indicate average of five random fields ± SEM.

### Three Dimensional Invasion Assays

Invasion assays were also performed using iuvo 3D-ICC slides (Bellbrook Labs). On day −1, channels were filled with diluted phenol red free matrigel (BD Biosciences) mixed with Texas Red labeled gelatin. Matrigel was polymerized at 37°C for 90 minutes; complete media was added to the right side port and media with 5 ng/ml EGF was added to the left port. Slide was incubated at 37°C overnight. Flow sorted HDF cells transfected with GFP-empty vector or WT palladin-GFP were labeled with QTracker585 (Invitrogen) as per manufacturer's instructions, trypsinized, and counted with a hemacytometer. 3,250 cells were loaded into the right side port and fresh media+EGF added to the left side port. Slide was incubated at ∼30° angle to allow for attachment of cells to the channel. 24 hours later, Panc1 cells were labeled with QTracker655 (Invitrogen) and 650 cells were added to the right side port. Cells were grown for 3 days and media changed daily. Images were taken using the Zeiss LSM 510 Meta confocal microscope using the 10× or 20× objectives and sequential scans with the 568 nm and 405 nm lasers. Images were exported from LSM Browser and analyzed with ImageJ.

### Invadopodia Assay

Coverslips were prepared as described previously [60] with modifications. In brief, coverslips were coated with Texas Red labeled gelatin prepared with FluoReporter kit (Molecular Probes) as per manufacturer's instructions. Gelatin was heated to 37°C, filtered through a 0.45 µm filter and spread onto coverslips. Excess gelatin was removed and coverslips were let stand at ≥60° angle for 30 minutes. Coverslips were incubated with cold glutaraldehyde, quenched with sodium borohydride, washed with PBS, sterilized with 70% ethanol, and incubated with serum-free media and then wounding conditioned media prior to cell plating. 10^5^ cells were plated onto coverslips and returned to 37°C for 18 hours. Coverslips were fixed and mounted onto slides as described above. Each transfection was tested multiple times. Shown are representative images.

### RhoA activation assay

Flow sorted GFP-empty vector or GFP-palladin expressing HDF cells were grown overnight on gelatin coated coverslips in wounding media as described above. RhoA G-LISA assay (Cytoskeleton) was performed as reported [61]. Shown is the average of six wells ± SD.

### Scanning electron microscopy

Samples were grown on coverslips and fixed at 37°C in fresh half strength Karnovsky's fixative and then overnight at 4°C. Specimens were rinsed with cacodylate buffer and post fixed with osmium tetroxide. Specimens were rinsed with distilled water, en bloc stained with 2% uranyl acetate for 45 minutes and dehydrated through a graded series of ethanols. Samples were critical point dried with a Samdri-PVT-3B CPD (Tousimis Research), mounted on aluminum stubs, sputter coated with gold-palladium (Polaron SEM Coating Unit, E 5100, Polaron Instruments Inc) and examined with a JEOL JSM 6300F field emission scanning electron microscope (JEOL, Tokyo, Japan) at an accelerating voltage of 15 kV. Shown are representative images.

### Proteomics Sample preparation

2×10^6^ cells were plated atop a transwell filter (Corning; 3 µm pores) over wounded HDF cells. Sixteen hours after plating, protein lysates were prepared as described in cold RIPA buffer [62]. Lysates from equivalent numbers of cells expressing empty vector (SILAC labeled), or unlabeled WT or FX palladin were combined, acetone precipitated, and resuspended in 50 mM ammonium bicarbonate. The proteins were reduced with DTT, blocked with iodoacetimide, and digested with trypsin overnight. Samples were purified over a C18 column (Nest Group, Inc.), dried in a speed vacuum and stored at −20°C until mass spectrometric analysis.

### Western blot analysis

Protein lysates from equivalent numbers of cells were separated by SDS-PAGE and transferred to HybondP membrane using semi-dry transfer. Blots were blocked with 1× Superblock for 1 hour at RT, and then incubated with primary antibody diluted 1∶2000 in 0.5× Superblock overnight at 4°C. Blots were washed and incubated with HRP-conjugated goat anti-mouse or donkey anti-rabbit (Thermo) diluted 1∶2000 in 0.5× Superblock for 1 hour at RT. Blots were washed and protein bands visualized using the Storm Phosphoimager following incubation with ECL Plus (GE Life Sciences).

### Mass spectrometric analysis

The samples were analyzed using an LTQ-Orbitrap hybrid mass spectrometer (Thermo Fisher Scientific) coupled with nano-flow HPLC, which consists of a trap column (100 µm×1.5 cm) packed with Magic C18AQ resin (5 µm, 200 Å particles; Michrom Bioresources), followed by an analytical column (75 µm×27 cm) packed with Magic C18AQ resin (5 µm, 100 Å particles; Michrom Bioresources). The peptide samples were analyzed using a 90-minute non-linear gradient, starting at 5% acetonitrile with 0.1% formic acid (against water with 0.1% formic acid), changing to 7% over 2 minutes, then to 35% over 90 minutes with a flow rate at 300 nL/min. The mass spectrometry experiment consisted of a full MS scan in the Orbitrap followed by up to 5 MS/MS spectra acquisitions in the linear ion trap using collision induced dissociation. An exclusion time of 45 sec was used to enhance the interrogation of low abundance peptides.

### Proteomics data processing

The MS/MS data was searched against IPI human protein database using X!tandem algorithm. The assignment of peptide sequence was validated using PeptideProphet. Peptides with a probability score of 0.9 or above were selected for protein identification using ProteinProphet. The quantitative ratio of peptide/protein was calculated using Xpress software.

## Supporting Information

Figure S1 **Alpha actinin staining increases with pancreatic cancer progression.** **A**) Strong alpha-actinin staining was observed within the stromal compartment of pancreatic cancer (*right panel*) sections but dramatically reduced in normal tissue (*left panel*). Alpha-actinin is a binding partner of 90 kD palladin and is a known invadopodia protein. **B**) Plot indicates the mean IHC score ± SEM for normal pancreas (n = 20) or cancer (n = 21). Scoring guidelines are outlined in Table S1.
(TIF)

Figure S2 **Validation of up-regulated proteins identified by proteomics of “feet” from palladin-activated fibroblasts.** **A**) Invadopodia proteins: cofilin, cortactin, and profilin, are confirmed to be up-regulated by immunofluorescence (IF) (*top left panel, top right panel and bottom right panel, respectively*). Stem cell marker, nestin, is also up-regulated (*bottom left panel*). Green = palladin or empty vector; red = antibody (cofilin, cortactin, nestin, or profilin); blue = DAPI. **B**) Up-regulation of cortactin, profilin, and cathepsin D in lysates prepared from “feet” of palladin-activated fibroblasts compared to control fibroblasts with empty vector are demonstrated in the Western blot.
(TIF)

Table S1 **Semi-quantitative scoring guidelines for immunohistochemical staining of pancreas tissue sections.** Shown are semi-quantitative scoring guidelines for immunohistochemical staining of pancreas tissue sections. Results were scored as diffuse or focal and were graded semi-quantitatively for intensity of staining from 0 = no staining to 4+ = the most intense staining. The tissues that were stained included pre-cancerous low and high-grade dysplasia, cancer and normal pancreas. Scoring systems for periductal or lesional stroma and parenchymal stroma are presented. Parenchymal stroma designates stroma which is not solely associated with a duct or lesion and includes intralobular stroma between acini, as well as interlobular stroma and confluent areas of fibrosis.
(DOC)

Table S2 **Proteomics analysis of “feet” from palladin-activated fibroblasts.** Shown are the results of proteomics analysis of pseudopodia. The IPI protein identifier, gene symbol, description and ratios for fibroblasts transfected with wildtype (WT) or Family X mutant (FX) palladin relative to empty vector (EV). The total spectral counts reflect the number of peptides identified. Samples with no ratio shown but one spectral count reflect that the protein was present in the sample but could not be quantified. If no spectral counts were shown, the protein was absent or undetectable.
(DOC)
