# Supplementary figures and images for: Arousal of Cancer-Associated Stroma: Overexpression of Palladin Activates Fibroblasts to Promote Tumor Invasion
Source: PLoS One. 2012 Jan 23;7(1):e30219. doi: 10.1371/journal.pone.0030219 (PMC3264580; doi:10.1371/journal.pone.0030219)

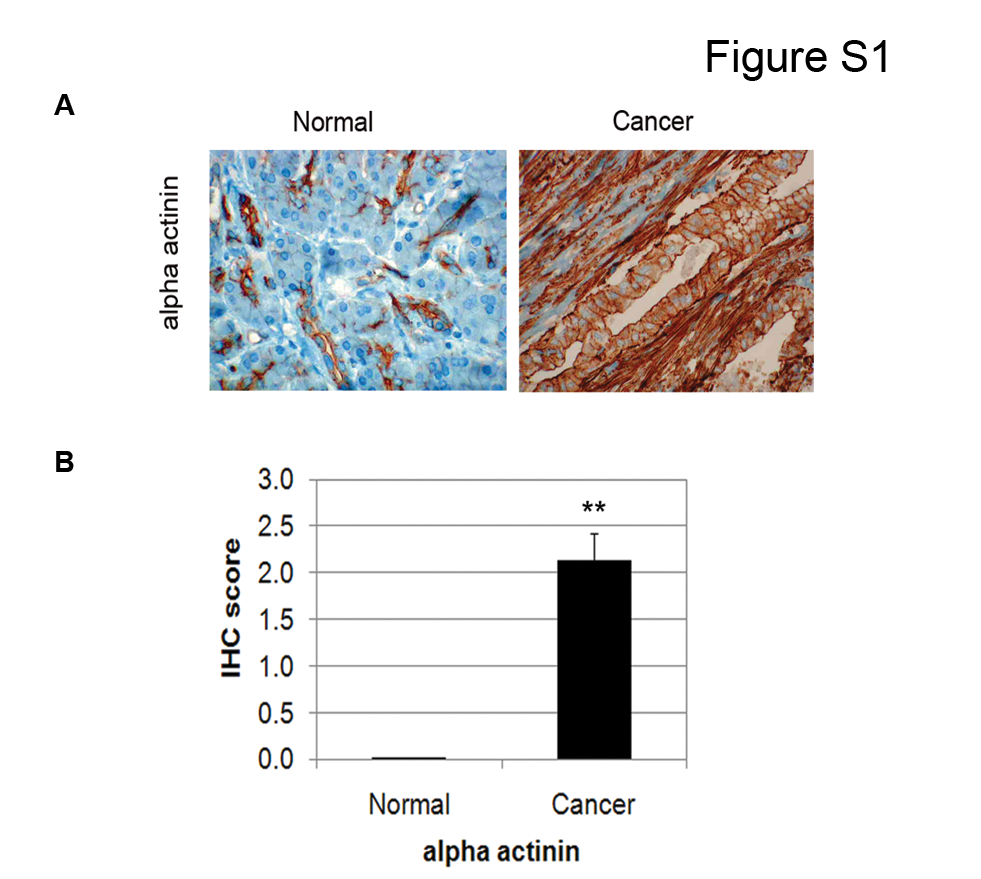

Supplement: Figure S1 — Alpha actinin staining increases with pancreatic cancer progression. A) Strong alpha-actinin staining was observed within the stromal compartment of pancreatic cancer (right panel) sections but dramatically reduced in normal tissue (left panel). Alpha-actinin is a binding partner of 90 kD palladin and is a known invadopodia protein. B) Plot indicates the mean IHC score ± SEM for normal pancreas (n = 20) or cancer (n = 21). Scoring guidelines are outlined in Table S1. (TIF) [file pone.0030219.s001.tif]

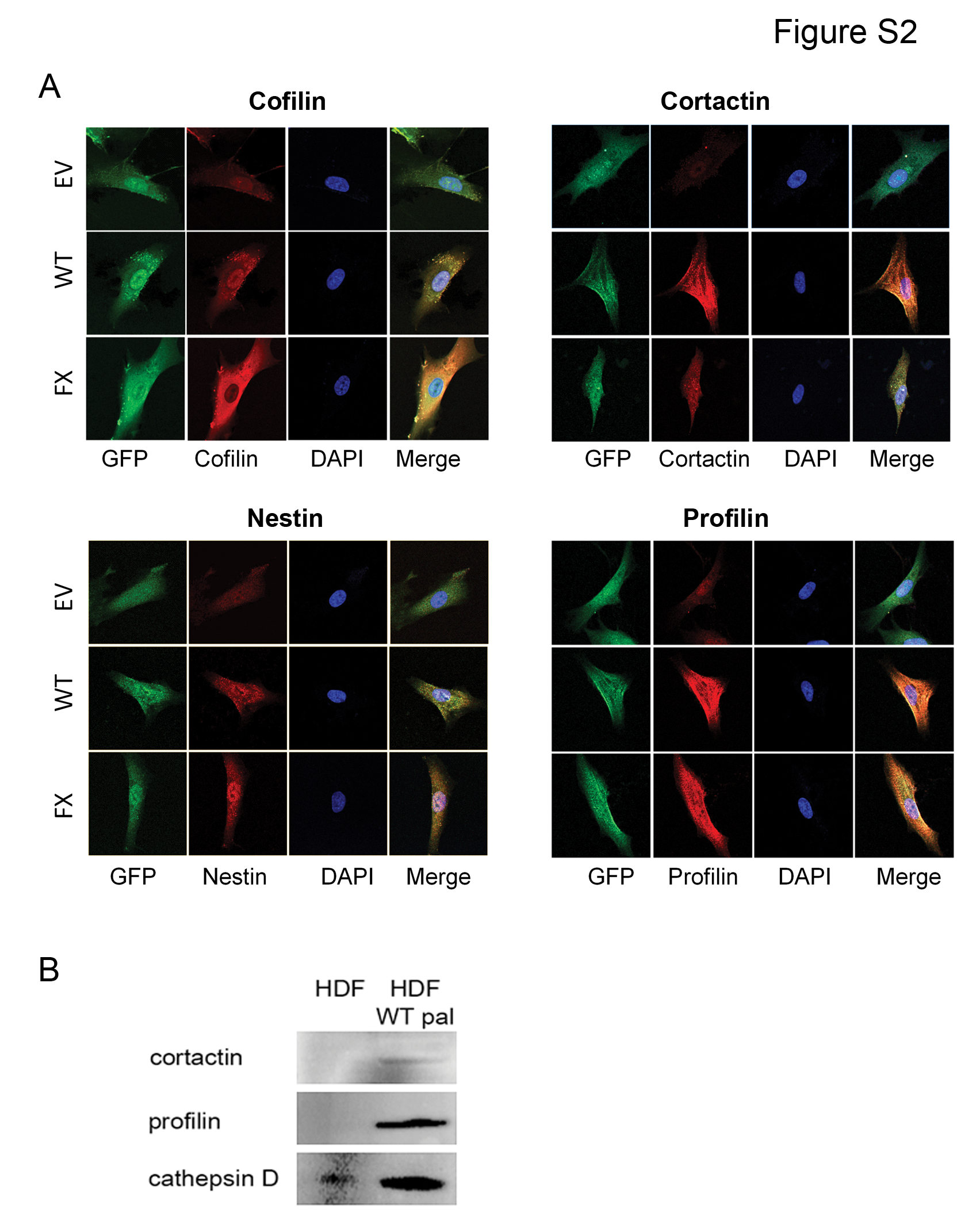

Supplement: Figure S2 — Validation of up-regulated proteins identified by proteomics of “feet” from palladin-activated fibroblasts. A) Invadopodia proteins: cofilin, cortactin, and profilin, are confirmed to be up-regulated by immunofluorescence (IF) (top left panel, top right panel and bottom right panel, respectively). Stem cell marker, nestin, is also up-regulated (bottom left panel). Green = palladin or empty vector; red = antibody (cofilin, cortactin, nestin, or profilin); blue = DAPI. B) Up-regulation of cortactin, profilin, and cathepsin D in lysates prepared from “feet” of palladin-activated fibroblasts compared to control fibroblasts with empty vector are demonstrated in the Western blot. (TIF) [file pone.0030219.s002.tif]
