# Supplementary material for: Arousal of Cancer-Associated Stroma: Overexpression of Palladin Activates Fibroblasts to Promote Tumor Invasion
Source: PLoS One. 2012 Jan 23;7(1):e30219. doi: 10.1371/journal.pone.0030219 (PMC3264580; doi:10.1371/journal.pone.0030219)
Supplement: Table S1 — Semi-quantitative scoring guidelines for immunohistochemical staining of pancreas tissue sections. Shown are semi-quantitative scoring guidelines for immunohistochemical staining of pancreas tissue sections. Results were scored as diffuse or focal and were graded semi-quantitatively for intensity of staining from 0 = no staining to 4+ = the most intense staining. The tissues that were stained included pre-cancerous low and high-grade dysplasia, cancer and normal pancreas. Scoring systems for periductal or lesional stroma and parenchymal stroma are presented. Parenchymal stroma designates stroma which is not solely associated with a duct or lesion and includes intralobular stroma between acini, as well as interlobular stroma and confluent areas of fibrosis. (DOC) [file pone.0030219.s003.doc]

Table S1. IHC scoring systems

| Scoring system for periductal or lesional stroma | |
| --- | --- |
| Score 0 | Absent staining |
| Score 1 | Staining limited to immediate vicinity of duct or lesion (less than 20 micron radial distance) |
| Score 2 | Staining of stroma adjacent to duct or lesion (approximately 20-60 micron radial distance) |
| Score 3 | Staining extends to stroma distant from duct or lesion (60 micron radial distance) but does not qualify for diffuse |
| Score 4 | Diffuse staining (at least 80% of stroma in the core involved) |

| Scoring system for parenchymal stroma | |
| --- | --- |
| Score 0 | Absent staining |
| Score 1 | Most fascicles of positively-staining stroma measure less than 20 microns. |
| Score 2 | Some fascicles of positively-staining stroma measure between approximately 20 microns and 60 microns. |
| Score 3 | Some fascicles of positively-staining stroma are broader than 60 microns. |
| Score 4 | Diffuse staining as defined above. |
