# Supplementary material for: Arousal of Cancer-Associated Stroma: Overexpression of Palladin Activates Fibroblasts to Promote Tumor Invasion
Source: PLoS One. 2012 Jan 23;7(1):e30219. doi: 10.1371/journal.pone.0030219 (PMC3264580; doi:10.1371/journal.pone.0030219)
Supplement: Table S2 — Proteomics analysis of “feet” from palladin-activated fibroblasts. Shown are the results of proteomics analysis of pseudopodia. The IPI protein identifier, gene symbol, description and ratios for fibroblasts transfected with wildtype (WT) or Family X mutant (FX) palladin relative to empty vector (EV). The total spectral counts reflect the number of peptides identified. Samples with no ratio shown but one spectral count reflect that the protein was present in the sample but could not be quantified. If no spectral counts were shown, the protein was absent or undetectable. (DOC) [file pone.0030219.s004.doc]

Table S2: Results of proteomics analysis of pseudopodia.

The IPI protein identifier, gene symbol, description and ratios for fibroblasts transfect with Palladin WT relative to EV and fibroblasts transfected with Palladin FX relative to EV are shown. The total spectral counts reflect the number of peptides which were identified in the sample. Samples with no ratio shown but one spectral count reflect that the protein was present in the sample but could not be quantitated. If no spectral counts were shown, the protein was absent or undetectable.

| **Protein ID** | **Gene Symbol** | **Description** | **Ratio WT/EV** | **Total Spectra Counts** | **Ratio FX/EV** | **Total Spectra Counts** |
| --- | --- | --- | --- | --- | --- | --- |
| IPI00024254 | IFIT3 | INTERFERON-INDUCED PROTEIN WITH TETRATRICOPEPTIDE REPEATS 3. | 34.50 | 1 | 19.26 | 1 |
| IPI00478003 | A2M | ALPHA-2-MACROGLOBULIN. | 21.93 | 4 | ND | 4 |
| IPI00022434;IPI00745872;IPI00908876 | ALB | ISOFORM 1 OF SERUM ALBUMIN. | 16.10 | 4 | 23.87 | 6 |
| IPI00021841;IPI00853525 | APOA1 | APOLIPOPROTEIN A-I. | 14.59 | 2 | 11.84 | 4 |
| IPI00010800 | NES | NESTIN. | 11.30 | 6 |  | 3 |
| IPI00848226 | GNB2L1 | GUANINE NUCLEOTIDE-BINDING PROTEIN SUBUNIT BETA-2-LIKE 1. | 9.49 | 3 | 2.28 | 1 |
| IPI00021700 | PCNA | PROLIFERATING CELL NUCLEAR ANTIGEN. | 8.58 | 2 | 8.07 | 1 |
| IPI00013881;IPI00026230;IPI00479191 | HNRNPH1;RPL36AP37;HNRNPH2 | HETEROGENEOUS NUCLEAR RIBONUCLEOPROTEIN H.; HETEROGENEOUS NUCLEAR RIBONUCLEOPROTEIN H2. | 7.33 | 3 | 1.38 | 2 |
| IPI00106668;IPI00303882;IPI00943550 | PLIN3 | MANNOSE 6 PHOSPHATE RECEPTOR BINDING PROTEIN 1 ISOFORM. | 7.05 | 4 | ND | 2 |
| IPI00216587;IPI00645201 | RPS8 | 40S RIBOSOMAL PROTEIN S8. | 7.00 | 2 | 5.16 | 2 |
| IPI00011253 | RPS3 | 40S RIBOSOMAL PROTEIN S3. | 6.77 | 2 | 6.71 | 4 |
| IPI00009904 | PDIA4 | PROTEIN DISULFIDE-ISOMERASE A4. | 6.33 | 3 | 3.71 | 6 |
| IPI00418471 | VIM | VIMENTIN. | 6.24 | 68 | 4.77 | 30 |
| IPI00419585;IPI00910407;IPI00925411;IPI00925747 | PPIA | PEPTIDYL-PROLYL CIS-TRANS ISOMERASE A. | 5.93 | 2 | 4.00 | 3 |
| IPI00016832;IPI00472442;IPI00871889 | PSMA1 | ISOFORM OF PROTEASOME SUBUNIT ALPHA TYPE-1. | 5.82 | 3 | 0.25 | 1 |
| IPI00299571;IPI00644989 | PDIA6 | ISOFORM OF PROTEIN DISULFIDE-ISOMERASE A6. | 5.60 | 3 | 0.51 | 4 |
| IPI00007188 | SLC25A5 | ADP/ATP TRANSLOCASE 2. | 5.56 | 3 | 3.25 | 1 |
| IPI00465028;IPI00797270 | TPI1;RCTPI1 | TRIOSEPHOSPHATE ISOMERASE 1 ISOFORM. | 5.47 | 8 | 4.92 | 9 |
| IPI00026570;IPI00872879 | COX7A2 | CYTOCHROME C OXIDASE POLYPEPTIDE 7A2. | 5.44 | 2 | 2.64 | 1 |
| IPI00290770;IPI00552715;IPI00553185 | CCT3 | CHAPERONIN CONTAINING TCP1, SUBUNIT 3 ISOFORM B. | 5.40 | 2 | ND | 1 |
| IPI00013122 | CDC37 | HSP90 CO-CHAPERONE CDC37. | 5.32 | 2 | 4.24 | 1 |
| IPI00026154;IPI00792916;IPI00829824;IPI00943074 | PRKCSH | PROTEIN KINASE C SUBSTRATE 80K-H ISOFORM 2. | 5.25 | 2 | 1.85 | 1 |
| IPI00219301 | MARCKS | MYRISTOYLATED ALANINE-RICH C-KINASE SUBSTRATE. | 5.23 | 2 | 3.69 | 3 |
| IPI00026314;IPI00646773;IPI00647556 | GSN | ISOFORM OF GELSOLIN. | 5.13 | 7 | 3.56 | 6 |
| IPI00072377;IPI00301311;IPI00735319;IPI00844040;IPI00896523;IPI00917753 | SET;LOC646817 | ISOFORM OF PROTEIN SET. | 5.13 | 3 | 2.61 | 1 |
| IPI00550363;IPI00644531;IPI00647915 | TAGLN2 | TRANSGELIN-2. | 5.03 | 7 | 3.17 | 3 |
| IPI00000874;IPI00640741 | PRDX1 | PEROXIREDOXIN-1. | 4.94 | 2 | 5.32 | 2 |
| IPI00018146 | YWHAQ | 14-3-3 PROTEIN THETA. | 4.92 | 7 | 2.14 | 5 |
| IPI00010896 | CLIC1 | CHLORIDE INTRACELLULAR CHANNEL PROTEIN 1. | 4.90 | 5 | 3.01 | 8 |
| IPI00021439;IPI00021440;IPI00894365;IPI00894498;IPI00930226 | ACTB;ACTG1;ACTB;ACTG1 | ACTIN, CYTOPLASMIC 1; ACTIN, CYTOPLASMIC 2. | 4.86 | 16 | 3.76 | 20 |
| IPI00216691 | PFN1 | PROFILIN-1. | 4.86 | 11 | 2.98 | 15 |
| IPI00335168;IPI00413922;IPI00789605;IPI00793089;IPI00795576;IPI00795944;IPI00796366;IPI00796500;IPI00797626;IPI00930599 | MYL6B;MYL6;MYL6B;MYL6 | ISOFORM NON-MUSCLE OF MYOSIN LIGHT POLYPEPTIDE 6.;ISOFORM SMOOTH MUSCLE OF MYOSIN LIGHT POLYPEPTIDE 6;CDNA FLJ56329, HIGHLY SIMILAR TO MYOSIN LIGHT POLYPEPTIDE 6. | 4.83 | 4 | ND | 1 |
| IPI00019376 | SEPT11 | CDNA FLJ53374, HIGHLY SIMILAR TO SEPTIN-11. | 4.80 | 2 | 2.20 | 2 |
| IPI00186290 | EEF2 | ELONGATION FACTOR 2. | 4.73 | 15 | 2.11 | 6 |
| IPI00218733 | SOD1 | SUPEROXIDE DISMUTASE [CU-ZN]. | 4.63 | 2 | 3.06 | 2 |
| IPI00026781 | FASN | FATTY ACID SYNTHASE. | 4.50 | 4 |  | 1 |
| IPI00217030 | RPS4X | 40S RIBOSOMAL PROTEIN S4, X ISOFORM. | 4.46 | 2 | 4.16 | 2 |
| IPI00219446 | PEBP1 | PHOSPHATIDYLETHANOLAMINE-BINDING PROTEIN 1. | 4.46 | 2 | 2.25 | 5 |
| IPI00025491 | EIF4A1;SNORA67 | EUKARYOTIC INITIATION FACTOR 4A-I. | 4.42 | 1 | 3.73 | 5 |
| IPI00012048;IPI00375531 | NME1 | ISOFORM OF NUCLEOSIDE DIPHOSPHATE KINASE A. | 4.41 | 5 | 3.01 | 3 |
| IPI00291175;IPI00307162 | VCL | ISOFORM 1 OF VINCULIN; ISOFORM 2 OF VINCULIN. | 4.34 | 13 | 2.83 | 12 |
| IPI00179330;IPI00418813;IPI00456429;IPI00654754;IPI00719280;IPI00784990;IPI00789107;IPI00789823;IPI00790633;IPI00792139;IPI00792712;IPI00793330;IPI00793729;IPI00793810;IPI00794205;IPI00794211;IPI00794925;IPI00795527;IPI00796007;IPI00796600;IPI00797400;IPI00798127;IPI00798155 | UBC;UBB;RPS27A;UBA52 | UBIQUITIN AND RIBOSOMAL PROTEIN S27A PRECURSOR;UBIQUITIN AND RIBOSOMAL PROTEIN L40 PRECURSOR;RPS27A PROTEIN;UBIQUITIN B PRECURSOR;UBIQUITIN C SPLICE VARIANT;UBIQUITIN C;39 KDA PROTEIN;CDNA FLJ51326, HIGHLY SIMILAR TO HOMO SAPIENS UBIQUITIN B (UBB), MRNA;UBIQUITIN. | 4.25 | 2 | 2.98 | 1 |
| IPI00000875;IPI00909534;IPI00937615 | EEF1G;TUT1 | HIGHLY SIMILAR TO ELONGATION FACTOR 1-GAMMA; ELONGATION FACTOR 1-GAMMA. | 4.21 | 5 | 4.08 | 1 |
| IPI00075248;IPI00794543;IPI00916600;IPI00916768 | CALM3;CALM2;CALM1 | CALMODULIN. | 4.19 | 3 | 2.59 | 7 |
| IPI00024175;IPI00218372;IPI00647380 | PSMA7 | ISOFORM 1 OF PROTEASOME SUBUNIT ALPHA TYPE-7; ISOFORM 2 OF PROTEASOME SUBUNIT ALPHA TYPE-7; PROTEASOME (PROSOME, MACROPAIN) SUBUNIT, ALPHA TYPE, 7. | 4.17 | 2 | 3.48 | 2 |
| IPI00021263 | YWHAZ | 14-3-3 PROTEIN ZETA/DELTA. | 4.15 | 9 | 2.41 | 7 |
| IPI00329801;IPI00872379 | ANXA5 | ANNEXIN A5. | 4.12 | 16 | 2.54 | 17 |
| IPI00013452 | EPRS | BIFUNCTIONAL AMINOACYL-TRNA SYNTHETASE. | 4.11 | 2 | 2.58 | 1 |
| IPI00000873;IPI00646425;IPI00793323;IPI00893211;IPI00893333;IPI00893918 | VARS | VALYL-TRNA SYNTHETASE. | 4.10 | 2 |  | 1 |
| IPI00216049;IPI00216746;IPI00514561;IPI00807545;IPI00910458 | HNRNPK | ISOFORM 1 OF HETEROGENEOUS NUCLEAR RIBONUCLEOPROTEIN K; ISOFORM 2 OF HETEROGENEOUS NUCLEAR RIBONUCLEOPROTEIN K; HETEROGENEOUS NUCLEAR RIBONUCLEOPROTEIN K;ISOFORM 3 OF HETEROGENEOUS NUCLEAR RIBONUCLEOPROTEIN K;CDNA FLJ54552, HIGHLY SIMILAR TO HETEROGENEOUS NUCLEAR RIBONUCLEOPROTEIN K. | 4.03 | 5 | 4.96 | 2 |
| IPI00014516;IPI00218694;IPI00218695;IPI00218696;IPI00333771;IPI00816770;IPI00925125;IPI00926274 | CALD1 | ISOFORM 1 OF CALDESMON. | 4.01 | 6 | 1.25 | 6 |
| IPI00219757;IPI00793319 | GSTP1 | GLUTATHIONE S-TRANSFERASE P; PUTATIVE UNCHARACTERIZED PROTEIN GSTP1. | 4.01 | 5 | 3.45 | 5 |
| IPI00215719 | RPL18 | 60S RIBOSOMAL PROTEIN L18. | 4.00 | 1 | 3.29 | 3 |
| IPI00012011 | CFL1 | COFILIN-1. | 3.96 | 7 | 2.77 | 12 |
| IPI00019502 | MYH9 | ISOFORM 1 OF MYOSIN-9. | 3.89 | 44 | 1.91 | 23 |
| IPI00298547 | PARK7 | PROTEIN DJ-1. | 3.88 | 2 | 1.32 | 1 |
| IPI00410714 | HBA2;HBA1 | HEMOGLOBIN SUBUNIT ALPHA. | 3.85 | 6 | 35.61 | 3 |
| IPI00297779 | CCT2 | T-COMPLEX PROTEIN 1 SUBUNIT BETA. | 3.82 | 5 | ND | 1 |
| IPI00007765 | HSPA9 | STRESS-70 PROTEIN, MITOCHONDRIAL. | 3.79 | 10 | 2.35 | 10 |
| IPI00021405;IPI00216953 | LMNA | ISOFORM A OF LAMIN-A/C;ISOFORM ADELTA10 OF LAMIN-A/C. | 3.77 | 10 | 2.29 | 6 |
| IPI00414676 | HSP90AB1 | HEAT SHOCK PROTEIN HSP 90-BETA. | 3.76 | 13 | 3.54 | 8 |
| IPI00217561;IPI00217562;IPI00217563;IPI00293305;IPI00549336 | ITGB1 | ISOFORM BETA-1C OF INTEGRIN BETA-1; ISOFORM BETA-1C-2 OF INTEGRIN BETA-1; ISOFORM BETA-1A OF INTEGRIN BETA-1; ISOFORM BETA-1B OF INTEGRIN BETA-1; ISOFORM BETA-1D OF INTEGRIN BETA-1. | 3.74 | 8 | 3.49 | 2 |
| IPI00027230 | HSP90B1 | ENDOPLASMIN. | 3.74 | 10 | 2.73 | 7 |
| IPI00010810;IPI00792673;IPI00895865 | ETFA | ELECTRON TRANSFER FLAVOPROTEIN SUBUNIT ALPHA, MITOCHONDRIAL; ELECTRON TRANSFER FLAVOPROTEIN, ALPHA POLYPEPTIDE ISOFORM B. | 3.73 | 2 | 2.61 | 1 |
| IPI00456969 | DYNC1H1 | CYTOPLASMIC DYNEIN 1 HEAVY CHAIN 1. | 3.69 | 5 | ND | 1 |
| IPI00027107 | TUFM | TU TRANSLATION ELONGATION FACTOR, MITOCHONDRIAL PRECURSOR. | 3.68 | 2 | ND | 2 |
| IPI00171903;IPI00383296 | HNRNPM | ISOFORM OF HETEROGENEOUS NUCLEAR RIBONUCLEOPROTEIN M. | 3.67 | 1 | 8.37 | 2 |
| IPI00024067;IPI00455383 | CLTC | ISOFORM 1 OF CLATHRIN HEAVY CHAIN 1. | 3.66 | 15 | 3.27 | 5 |
| IPI00220642;IPI00910779 | YWHAG | 14-3-3 PROTEIN GAMMA. | 3.63 | 4 | 3.10 | 4 |
| IPI00479186 | PKM2 | ISOFORM M2 OF PYRUVATE KINASE ISOZYMES M1/M2. | 3.61 | 34 | 3.14 | 28 |
| IPI00000816 | YWHAE | 14-3-3 PROTEIN EPSILON. | 3.57 | 7 | 3.59 | 2 |
| IPI00031522 | HADHA | TRIFUNCTIONAL ENZYME SUBUNIT ALPHA, MITOCHONDRIAL. | 3.57 | 4 | 0.88 | 3 |
| IPI00302592;IPI00333541;IPI00644576 | FLNA | ISOFORM OF FILAMIN-A. | 3.53 | 37 | 2.42 | 19 |
| IPI00006482;IPI00646182 | ATP1A1 | ISOFORM LONG OF SODIUM/POTASSIUM-TRANSPORTING ATPASE SUBUNIT ALPHA-1. | 3.51 | 5 | 0.96 | 3 |
| IPI00216319 | YWHAH | 14-3-3 PROTEIN ETA. | 3.50 | 4 | ND | 1 |
| IPI00013808 | ACTN4 | ALPHA-ACTININ-4. | 3.49 | 20 | 2.45 | 7 |
| IPI00382470;IPI00784295 | HSP90AA1 | ISOFORM OF HEAT SHOCK PROTEIN HSP 90-ALPHA. | 3.49 | 10 | 3.74 | 10 |
| IPI00010796 | P4HB | PROTEIN DISULFIDE-ISOMERASE. | 3.45 | 7 | 3.00 | 3 |
| IPI00022774 | VCP | TRANSITIONAL ENDOPLASMIC RETICULUM ATPASE. | 3.43 | 4 | 1.76 | 7 |
| IPI00008529 | RPLP2 | 60S ACIDIC RIBOSOMAL PROTEIN P2. | 3.41 | 6 | 2.21 | 6 |
| IPI00021048;IPI00216268;IPI00216269;IPI00385318;IPI00645867 | MYOF | ISOFORM OF MYOFERLIN. | 3.38 | 8 | 1.27 | 1 |
| IPI00216318;IPI00759832 | YWHAB | ISOFORM OF 14-3-3 PROTEIN BETA/ALPHA. | 3.38 | 8 | 2.72 | 9 |
| IPI00470528;IPI00790569;IPI00925058;IPI00926202;IPI00927715 | RPL15 | 60S RIBOSOMAL PROTEIN L15. | 3.36 | 2 | 0.91 | 1 |
| IPI00008274 | CAP1 | ISOFORM 1 OF ADENYLYL CYCLASE-ASSOCIATED PROTEIN 1. | 3.32 | 7 | 2.25 | 4 |
| IPI00000105 | MVP | MAJOR VAULT PROTEIN | 3.31 | 3 | 11.47 | 2 |
| IPI00295386 | CBR1 | CARBONYL REDUCTASE [NADPH] 1. | 3.31 | 3 | 1.08 | 2 |
| IPI00016342 | RAB7A | RAS-RELATED PROTEIN RAB-7A. | 3.30 | 3 | 2.25 | 4 |
| IPI00645078 | UBA1 | UBIQUITIN-LIKE MODIFIER-ACTIVATING ENZYME 1. | 3.27 | 8 | 1.04 | 5 |
| IPI00939159 | CAP1 | ADENYLYL CYCLASE-ASSOCIATED PROTEIN. | 3.26 | 8 | 2.22 | 4 |
| IPI00453476;IPI00549725 | PGAM1 | PHOSPHOGLYCERATE MUTASE 1. | 3.25 | 4 | ND | 1 |
| IPI00295400 | WARS | ISOFORM 1 OF TRYPTOPHANYL-TRNA SYNTHETASE, CYTOPLASMIC. | 3.25 | 2 | 2.83 | 3 |
| IPI00013744 | ITGA2 | INTEGRIN ALPHA-2. | 3.23 | 9 | 1.66 | 6 |
| IPI00010779 | TPM4 | ISOFORM 1 OF TROPOMYOSIN ALPHA-4 CHAIN. | 3.22 | 5 | 2.66 | 4 |
| IPI00911039 | HSPA1B;HSPA1A | CDNA FLJ54408, HIGHLY SIMILAR TO HEAT SHOCK 70 KDA PROTEIN 1. | 3.20 | 7 | ND | 5 |
| IPI00017726 | HSD17B10 | ISOFORM 1 OF 3-HYDROXYACYL-COA DEHYDROGENASE TYPE-2. | 3.19 | 1 | 6.01 | 2 |
| IPI00302925;IPI00784090 | CCT8 | T-COMPLEX PROTEIN 1 SUBUNIT THETA. | 3.14 | 1 | 1.64 | 2 |
| IPI00010418;IPI00743335;IPI00791026;IPI00829992 | MYO1C | ISOFORM 2 OF MYOSIN-IC; ISOFORM 1 OF MYOSIN-IC; ISOFORM 3 OF MYOSIN-IC. | 3.13 | 4 | 3.33 | 1 |
| IPI00218918 | ANXA1 | ANNEXIN A1. | 3.10 | 10 | 2.89 | 11 |
| IPI00220327 | KRT1 | KERATIN, TYPE II CYTOSKELETAL 1. | 3.09 | 7 | 9.68 | 3 |
| IPI00020599 | CALR | CALRETICULIN. | 3.01 | 9 | 2.15 | 10 |
| IPI00141318;IPI00604713;IPI00883950;IPI00939370 | CKAP4 | ISOFORM 1 OF CYTOSKELETON-ASSOCIATED PROTEIN 4. | 2.98 | 9 | 3.77 | 10 |
| IPI00843975;IPI00872684 | EZR | EZRIN; 69 KDA PROTEIN. | 2.95 | 3 | 2.04 | 5 |
| IPI00025318 | SH3BGRL | SH3 DOMAIN-BINDING GLUTAMIC ACID-RICH-LIKE PROTEIN. | 2.93 | 2 | 8.23 | 1 |
| IPI00221226 | ANXA6 | ANNEXIN A6. | 2.91 | 9 | 2.89 | 6 |
| IPI00298994 | TLN1 | TALIN-1. | 2.86 | 11 | 1.77 | 10 |
| IPI00219025 | GLRX | GLUTAREDOXIN-1. | 2.86 | 2 | 1.36 | 2 |
| IPI00007752 | TUBB2C | TUBULIN BETA-2C CHAIN. | 2.81 | 7 | 4.08 | 18 |
| IPI00465248 | ENO1 | ISOFORM ALPHA-ENOLASE OF ALPHA-ENOLASE. | 2.81 | 26 | 2.69 | 30 |
| IPI00014230;IPI00796075 | C1QBP | COMPLEMENT COMPONENT 1 Q SUBCOMPONENT-BINDING PROTEIN, MITOCHONDRIAL. | 2.80 | 3 | 1.87 | 1 |
| IPI00386533;IPI00442069;IPI00479262;IPI00552639;IPI00759560;IPI00796985;IPI00798400;IPI00924828;IPI00925263;IPI00925413;IPI00926411;IPI00927510;IPI00927765 | EIF4G1 | EUKARYOTIC TRANSLATION INITIATION FACTOR 4 GAMMA, 1 ISOFORM 4; ISOFORM B OF EUKARYOTIC TRANSLATION INITIATION FACTOR 4 GAMMA 1; ISOFORM 1 OF EUKARYOTIC TRANSLATION INITIATION FACTOR 4 GAMMA 1; EIF4G1 PROTEIN; 29 KDA PROTEIN.; EUKARYOTIC TRANSLATION INITIATION FACTOR 4 GAMMA, 1 ISOFORM 3; 1; EUKARYOTIC TRANSLATION INITIATION FACTOR 4 GAMMA, 1 ISOFORM 2; ISOFORM D OF EUKARYOTIC TRANSLATION INITIATION FACTOR 4 GAMMA 1; ISOFORM E OF EUKARYOTIC TRANSLATION INITIATION FACTOR 4 GAMMA 1. | 2.79 | 2 | ND | 1 |
| IPI00009634 | SQRDL | SULFIDE: QUINONE OXIDOREDUCTASE, MITOCHONDRIAL. | 2.79 | 5 | 4.41 | 3 |
| IPI00219219 | LGALS1 | GALECTIN-1. | 2.76 | 4 | 3.35 | 2 |
| IPI00003865 | HSPA8 | ISOFORM 1 OF HEAT SHOCK COGNATE 71 KDA PROTEIN. | 2.72 | 20 | 3.08 | 29 |
| IPI00032140 | SERPINH1 | SERPIN H1. | 2.71 | 6 | 5.26 | 7 |
| IPI00215743;IPI00220967;IPI00744135;IPI00856098 | RRBP1 | ISOFORM 3 OF RIBOSOME-BINDING PROTEIN 1; ISOFORM 1 OF RIBOSOME-BINDING PROTEIN 1; ISOFORM 2 OF RIBOSOME-BINDING PROTEIN 1; P180/RIBOSOME RECEPTOR. | 2.70 | 7 | 4.99 | 2 |
| IPI00018140;IPI00402182;IPI00402183;IPI00402184;IPI00402185;IPI00930205 | SYNCRIP | ISOFORM 1 OF HETEROGENEOUS NUCLEAR RIBONUCLEOPROTEIN Q; ISOFORM 2 OF HETEROGENEOUS NUCLEAR RIBONUCLEOPROTEIN Q; ISOFORM 3 OF HETEROGENEOUS NUCLEAR RIBONUCLEOPROTEIN Q; ISOFORM 4 OF HETEROGENEOUS NUCLEAR RIBONUCLEOPROTEIN Q; SYNAPTOTAGMIN BINDING, CYTOPLASMIC RNA INTERACTING PROTEIN ISOFORM 2; SYNAPTOTAGMIN BINDING, CYTOPLASMIC RNA INTERACTING PROTEIN ISOFORM 5. | 2.64 | 2 | 2.37 | 1 |
| IPI00646304 | PPIB | PEPTIDYL-PROLYL CIS-TRANS ISOMERASE B. | 2.60 | 4 | 4.23 | 7 |
| IPI00156689 | VAT1 | SYNAPTIC VESICLE MEMBRANE PROTEIN VAT-1 HOMOLOG. | 2.56 | 4 | 1.97 | 5 |
| IPI00008485 | ACO1 | CYTOPLASMIC ACONITATE HYDRATASE. | 2.54 | 3 | 2.22 | 2 |
| IPI00012007 | AHCY | ADENOSYLHOMOCYSTEINASE. | 2.50 | 2 |  | 1 |
| IPI00021812 | AHNAK | NEUROBLAST DIFFERENTIATION-ASSOCIATED PROTEIN AHNAK. | 2.45 | 19 | 1.41 | 20 |
| IPI00024933;IPI00868816 | RPL12;RPL12P38 | ISOFORM 1 OF 60S RIBOSOMAL PROTEIN L12. | 2.45 | 2 | 1.08 | 2 |
| IPI00003815;IPI00793767;IPI00794402;IPI00796541;IPI00872531 | ARHGDIA | RHO GDP-DISSOCIATION INHIBITOR 1. | 2.45 | 3 | 1.73 | 2 |
| IPI00479217;IPI00644079;IPI00644224;IPI00883857;IPI00915829 | HNRNPU | ISOFORM OF HETEROGENEOUS NUCLEAR RIBONUCLEOPROTEIN U. | 2.39 | 3 |  | 1 |
| IPI00291136 | COL6A1 | COLLAGEN ALPHA-1(VI) CHAIN. | 2.36 | 2 | 8.46 | 1 |
| IPI00024915;IPI00375306;IPI00759663 | PRDX5 | ISOFORM MITOCHONDRIAL OF PEROXIREDOXIN-5, MITOCHONDRIAL. | 2.36 | 3 | 2.39 | 1 |
| IPI00013894;IPI00479946 | STIP1 | STRESS-INDUCED-PHOSPHOPROTEIN 1. | 2.35 | 2 | 3.53 | 1 |
| IPI00000877;IPI00922127;IPI00922838 | HYOU1 | HYPOXIA UP-REGULATED PROTEIN 1. | 2.35 | 2 | 1.19 | 1 |
| IPI00003362 | HSPA5 | HSPA5 PROTEIN. | 2.35 | 17 | 2.77 | 16 |
| IPI00016801;IPI00027146 | GLUD1;GLUD2 | GLUTAMATE DEHYDROGENASE 1, MITOCHONDRIAL; GLUTAMATE DEHYDROGENASE 2, MITOCHONDRIAL. | 2.34 | 2 | 2.70 | 3 |
| IPI00014898 | PLEC1 | ISOFORM 1 OF PLECTIN-1. | 2.34 | 26 | 1.86 | 20 |
| IPI00007117 | SERPINB2 | PLASMINOGEN ACTIVATOR INHIBITOR 2. | 2.30 | 5 | 1.47 | 8 |
| IPI00221224 | ANPEP | AMINOPEPTIDASE N. | 2.26 | 3 | 2.06 | 3 |
| IPI00023048;IPI00064086;IPI00642971;IPI00789435 | EEF1D | ISOFORM OF ELONGATION FACTOR 1-DELTA. | 2.25 | 3 |  | 1 |
| IPI00440493 | ATP5A1 | ATP SYNTHASE SUBUNIT ALPHA, MITOCHONDRIAL. | 2.25 | 6 | 1.42 | 4 |
| IPI00011229 | CTSD | CATHEPSIN D. | 2.24 | 5 | 1.88 | 3 |
| IPI00024911 | ERP29 | ENDOPLASMIC RETICULUM PROTEIN ERP29. | 2.23 | 1 | 2.63 | 2 |
| IPI00479722;IPI00748256 | PSME1 | PROTEASOME ACTIVATOR COMPLEX SUBUNIT 1. | 2.22 | 2 | 5.21 | 1 |
| IPI00027497;IPI00908881;IPI00910781 | GPI | GLUCOSE-6-PHOSPHATE ISOMERASE. | 2.17 | 4 | 3.26 | 2 |
| IPI00013508;IPI00759776;IPI00909239;IPI00921118 | ACTN1 | ALPHA-ACTININ-1; ACTININ, ALPHA 1 ISOFORM A. | 2.16 | 26 | 2.31 | 12 |
| IPI00218342;IPI00794900 | MTHFD1 | C-1-TETRAHYDROFOLATE SYNTHASE, CYTOPLASMIC. | 2.16 | 2 | 2.26 | 1 |
| IPI00444262;IPI00604620 | NCL | CDNA FLJ45706 FIS, CLONE FEBRA2028457, HIGHLY SIMILAR TO NUCLEOLIN; NUCLEOLIN. | 2.12 | 4 | 3.06 | 2 |
| IPI00021766;IPI00298289;IPI00478442 | RTN4 | ISOFORM OF RETICULON-4. | 2.09 | 6 | ND | 2 |
| IPI00027463 | S100A6 | PROTEIN S100-A6. | 2.08 | 1 | 2.11 | 3 |
| IPI00643920;IPI00792641;IPI00793119;IPI00940673;IPI00942979 | TKT | TRANSKETOLASE ISOFORM 2. | 2.08 | 4 | 3.37 | 3 |
| IPI00793199;IPI00872780 | ANXA4 | ANNEXIN IV. | 2.06 | 4 |  | 1 |
| IPI00784154 | HSPD1 | 60 KDA HEAT SHOCK PROTEIN, MITOCHONDRIAL. | 2.06 | 8 | 5.09 | 4 |
| IPI00178352;IPI00413958 | FLNC | ISOFORM OF FILAMIN-C. | 2.06 | 12 | 2.45 | 7 |
| IPI00169383;IPI00909158;IPI00910974;IPI00916818 | PGK1 | PHOSPHOGLYCERATE KINASE 1. | 2.04 | 5 | ND | 1 |
| IPI00216298;IPI00552768 | TXN | THIOREDOXIN. | 2.03 | 3 | 1.64 | 1 |
| IPI00219217 | LDHB | L-LACTATE DEHYDROGENASE B CHAIN. | 1.97 | 5 | 2.18 | 5 |
| IPI00009342 | IQGAP1 | RAS GTPASE-ACTIVATING-LIKE PROTEIN IQGAP1. | 1.92 | 11 | 0.44 | 5 |
| IPI00306960 | NARS | ASPARAGINYL-TRNA SYNTHETASE, CYTOPLASMIC. | 1.89 | 3 | 2.40 | 3 |
| IPI00291006;IPI00924593 | MDH2 | MALATE DEHYDROGENASE, MITOCHONDRIAL. | 1.83 | 5 | 2.02 | 4 |
| IPI00465439;IPI00796333 | ALDOA | FRUCTOSE-BISPHOSPHATE ALDOLASE A. | 1.77 | 6 | 2.64 | 6 |
| IPI00011200 | PHGDH | D-3-PHOSPHOGLYCERATE DEHYDROGENASE. | 1.75 | 4 | 0.37 | 3 |
| IPI00413641 | AKR1B1 | ALDOSE REDUCTASE. | 1.70 | 6 | 3.70 | 3 |
| IPI00419237;IPI00789806;IPI00910169 | LAP3 | ISOFORM OF CYTOSOL AMINOPEPTIDASE. | 1.63 | 2 | ND | 1 |
| IPI00020984;IPI00941747 | CANX | CALNEXIN. | 1.58 | 5 | 2.85 | 3 |
| IPI00023748;IPI00797126;IPI00797259;IPI00909970 | NACA | NASCENT POLYPEPTIDE-ASSOCIATED COMPLEX SUBUNIT ALPHA. | 1.57 | 1 | 2.69 | 2 |
| IPI00031461;IPI00640006;IPI00940148 | GDI2 | GDP DISSOCIATION INHIBITOR 2 ISOFORM 2. | 1.54 | 3 | 1.68 | 2 |
| IPI00299024 | BASP1 | ISOFORM 1 OF BRAIN ACID SOLUBLE PROTEIN 1. | 1.51 | 6 | 4.23 | 7 |
| IPI00783097;IPI00915808 | GARS | GLYCYL-TRNA SYNTHETASE. | 1.50 | 3 | 1.71 | 2 |
| IPI00025252 | PDIA3 | PROTEIN DISULFIDE-ISOMERASE A3. | 1.31 | 6 | 3.64 | 5 |
| IPI00418169;IPI00455315 | ANXA2 | ISOFORM 2 OF ANNEXIN A2. | 1.28 | 8 | 2.13 | 17 |
| IPI00022228;IPI00443983;IPI00894287 | HDLBP | VIGILIN. | 1.25 | 2 | 3.37 | 1 |
| IPI00549343;IPI00553138;IPI00790248;IPI00791199 | VAMP3;VAMP2 | VESICLE-ASSOCIATED MEMBRANE PROTEIN 3; VESICLE-ASSOCIATED MEMBRANE PROTEIN 2. | 1.21 | 2 | 1.57 | 1 |
| IPI00027626 | CCT6A | T-COMPLEX PROTEIN 1 SUBUNIT ZETA. | 1.20 | 3 | 3.69 | 2 |
| IPI00140420 | SND1 | STAPHYLOCOCCAL NUCLEASE DOMAIN-CONTAINING PROTEIN 1. | 1.02 | 3 | 4.76 | 5 |
| IPI00026272;IPI00031562;IPI00081836;IPI00216456;IPI00216457;IPI00220855;IPI00255316;IPI00291764;IPI00339274;IPI00552873;IPI00930144 | HIST1H2AD;HIST1H2AI;HIST1H2AG;HIST1H2AM;HIST1H2AJ;HIST1H2AE;HIST1H2AB;HIST1H2AK;HIST1H2AL;HIST3H2A;HIST1H2AI;HIST1H2AG;HIST1H2AM;HIST1H2AH;HIST1H2AJ;HIST1H2AK;HIST1H2AL;HIST1H2AC;HIST2H2AA3;HIST2H2AA4;H2AFJ;HIST1H2AD;HIST2H2AC;HIST1H2AI;HIST1H2AG;HIST1H2AM;HIST1H2AJ;HIST1H2AK;HIST1H2AL | HISTONE H2A TYPE 1-B/E; HISTONE H2A TYPE 3; HISTONE H2A TYPE 1-H; HISTONE H2A TYPE 1-C; HISTONE H2A TYPE 2-A; ISOFORM 1 OF HISTONE H2A.J; HISTONE H2A TYPE 1-D; HISTONE H2A TYPE 1; HISTONE H2A TYPE 2-C; HISTONE H2A TYPE 1-J; HISTONE H2A. | 0.95 | 2 | 2.24 | 1 |
| IPI00299573;IPI00397676;IPI00478896 | RPL7A | 60S RIBOSOMAL PROTEIN L7A. | 0.90 | 2 | 2.56 | 3 |
| IPI00021290;IPI00394838;IPI00939422 | ACLY | ATP-CITRATE SYNTHASE. | 0.85 | 2 | 6.39 | 3 |
| IPI00031420 | UGDH | UDP-GLUCOSE 6-DEHYDROGENASE. | 0.67 | 2 | 2.25 | 3 |
| IPI00303476 | ATP5B | ATP SYNTHASE SUBUNIT BETA, MITOCHONDRIAL. | 0.45 | 8 | 1.42 | 5 |
| IPI00000190;IPI00657752 | CD81 | CD81 ANTIGEN | 0.43 | 1 | 2.57 | 3 |
| IPI00008530;IPI00556485 | RPLP0 | 60S ACIDIC RIBOSOMAL PROTEIN P0; RPLP0 PROTEIN. | 0.41 | 4 | 2.68 | 4 |
| IPI00218319;IPI00218320;IPI00382894;IPI00477649;IPI00479185;IPI00642042 | TPM3 | ISOFORM OF TROPOMYOSIN ALPHA-3 CHAIN. | 0.13 | 2 | 3.13 | 4 |
| IPI00375631 | ISG15 | INTERFERON-INDUCED 17 KDA PROTEIN. |  |  | 17.31 | 2 |
| IPI00000779;IPI00220631;IPI00220632;IPI00220634;IPI00220635;IPI00927866 | ADAM22 | ISOFORM OF DISINTEGRIN AND METALLOPROTEINASE DOMAIN-CONTAINING PROTEIN 22. |  |  | 16.56 | 1 |
| IPI00412977;IPI00455510;IPI00479214;IPI00827535;IPI00916480;IPI00916962;IPI00917228;IPI00917401 | PTMAP5;PTMA | PROTHYMOSIN ALPHA. |  |  | 12.77 | 3 |
| IPI00012268;IPI00926258;IPI00926410 | PSMD2 | 26S PROTEASOME NON-ATPASE REGULATORY SUBUNIT 2. |  |  | 12.59 | 2 |
| IPI00022443 | AFP | ALPHA-FETOPROTEIN. |  |  | 7.86 | 3 |
| IPI00302927;IPI00893358;IPI00921414 | ILK-2;CCT4 | T-COMPLEX PROTEIN 1 SUBUNIT DELTA. |  |  | 6.54 | 2 |
| IPI00410693;IPI00412714;IPI00470497;IPI00470498 | SERBP1 | ISOFORM OF PLASMINOGEN ACTIVATOR INHIBITOR 1 RNA-BINDING PROTEIN. |  |  | 6.45 | 2 |
| IPI00304612;IPI00398949;IPI00398983;IPI00432865;IPI00936705 | RPL13A;LOC100287887;RPL13AP25;RPL13AP3;LOC100290339;LOC100293761 | 60S RIBOSOMAL PROTEIN L13A. |  |  | 6.28 | 1 |
| IPI00221093;IPI00797968 | RPS17 | 40S RIBOSOMAL PROTEIN S17. |  |  | 6.11 | 2 |
| IPI00022418;IPI00339223;IPI00339224;IPI00339225;IPI00339226;IPI00339227;IPI00339228;IPI00339319;IPI00414283;IPI00479723;IPI00556632;IPI00845263;IPI00855777;IPI00855785;IPI00867588;IPI00873210 | FN1 | ISOFORM OF FIBRONECTIN. |  |  | 5.65 | 5 |
| IPI00217966;IPI00607708;IPI00910754;IPI00939286;IPI00947127 | LDHA | ISOFORM OF L-LACTATE DEHYDROGENASE A CHAIN. |  |  | 5.40 | 11 |
| IPI00291510;IPI00925196 | IMPDH2 | INOSINE-5'-MONOPHOSPHATE DEHYDROGENASE 2. |  |  | 5.30 | 2 |
| IPI00032449;IPI00032453;IPI00294834;IPI00396582;IPI00746217;IPI00909538;IPI00910043;IPI00921959;IPI00941148;IPI00944640;IPI00944646 | ASPH | ASPARTATE BETA-HYDROXYLASE ISOFORM C. |  |  | 5.16 | 3 |
| IPI00031812;IPI00643351 | YBX1 | NUCLEASE-SENSITIVE ELEMENT-BINDING PROTEIN 1. |  |  | 5.16 | 4 |
| IPI00031801;IPI00219147;IPI00219148;IPI00555698 | CSDA | ISOFORM 1 OF DNA-BINDING PROTEIN A. |  |  | 5.11 | 2 |
| IPI00215637 | DDX3X | ATP-DEPENDENT RNA HELICASE DDX3X. |  |  | 4.90 | 2 |
| IPI00008527 | RPLP1 | 60S ACIDIC RIBOSOMAL PROTEIN P1. |  |  | 4.87 | 4 |
| IPI00216694;IPI00947227 | PLS3 | PLASTIN-3. |  |  | 4.76 | 5 |
| IPI00028031;IPI00178744;IPI00937735 | ACADVL | HIGHLY SIMILAR TO VERY-LONG-CHAIN SPECIFIC ACYL- COADEHYDROGENASE, MITOCHONDRIAL. |  |  | 4.62 | 2 |
| IPI00031812;IPI00643351 | YBX1 | NUCLEASE-SENSITIVE ELEMENT-BINDING PROTEIN 1. |  |  | 4.55 | 5 |
| IPI00003881 | HNRNPF | HETEROGENEOUS NUCLEAR RIBONUCLEOPROTEIN F. |  |  | 4.42 | 6 |
| IPI00022200;IPI00072917;IPI00072918;IPI00220701;IPI00941465;IPI00946286 | COL6A3 | ISOFORM 1 OF COLLAGEN ALPHA-3(VI) CHAIN. |  |  | 4.20 | 7 |
| IPI00215918;IPI00792330 | ARF4 | ADP-RIBOSYLATION FACTOR 4. |  |  | 3.99 | 5 |
| IPI00246058;IPI00938079 | PDCD6IP | PROGRAMMED CELL DEATH 6-INTERACTING PROTEIN. |  |  | 3.97 | 2 |
| IPI00376798;IPI00647168;IPI00647674;IPI00746438;IPI00878431 | RPL11 | ISOFORM OF 60S RIBOSOMAL PROTEIN L11. |  |  | 3.93 | 2 |
| IPI00302688;IPI00479537;IPI00513761;IPI00550928;IPI00645630;IPI00941904 | ECHDC1 | ISOFORM OF ENOYL-COA HYDRATASE DOMAIN-CONTAINING PROTEIN 1. |  |  | 3.90 | 1 |
| IPI00010720 | CCT5 | T-COMPLEX PROTEIN 1 SUBUNIT EPSILON. |  |  | 3.88 | 3 |
| IPI00012750;IPI00903251 | RPS25 | 40S RIBOSOMAL PROTEIN S25. |  |  | 3.88 | 2 |
| IPI00218343 | TUBA1C | TUBULIN ALPHA-1C CHAIN. |  |  | 3.83 | 22 |
| IPI00021304 | KRT2 | KERATIN, TYPE II CYTOSKELETAL 2 EPIDERMAL. |  |  | 3.68 | 2 |
| IPI00016786;IPI00909484;IPI00910473 | CDC42 | ISOFORM 2 OF CELL DIVISION CONTROL PROTEIN 42 HOMOLOG. |  |  | 3.60 | 5 |
| IPI00032826;IPI00168839;IPI00218038;IPI00847961;IPI00892521;IPI00892883 | ST13;FAM10A5;FAM10A4 | HSC70-INTERACTING PROTEIN; PROTEIN FAM10A5; PROTEIN FAM10A4; ST13 PROTEIN; PROTEIN; SUPPRESSION OF TUMORIGENICITY 13. |  |  | 3.60 | 2 |
| IPI00011654 | TUBB | TUBULIN BETA CHAIN. |  |  | 3.59 | 26 |
| IPI00033494;IPI00220573;IPI00604523;IPI00719669 | MYL12B;MYL12A | MYOSIN REGULATORY LIGHT CHAIN 12B; MYOSIN REGULATORY LIGHT CHAIN 12A. |  |  | 3.51 | 2 |
| IPI00030929;IPI00220278 | MYL9 | MYOSIN REGULATORY LIGHT CHAIN 9 ISOFORM B. |  |  | 3.51 | 2 |
| IPI00478231;IPI00926710;IPI00927114 | RHOA | TRANSFORMING PROTEIN RHOA. |  |  | 3.50 | 2 |
| IPI00006935;IPI00376005;IPI00411704;IPI00855924;IPI00945644;IPI00946502;IPI00946642 | EIF5A2;EIF5A;EIF5A;EIF5AL1 | EUKARYOTIC TRANSLATION INITIATION FACTOR 5A-2; ISOFORM 2 OF EUKARYOTIC TRANSLATION INITIATION FACTOR 5A-1; ISOFORM 1 OF EUKARYOTIC TRANSLATION INITIATION FACTOR 5A-1; EUKARYOTIC TRANSLATION INITIATION FACTOR 5A-1-LIKE. |  |  | 3.47 | 2 |
| IPI00168184;IPI00554737;IPI00798040;IPI00908543;IPI00910732 | PPP2R1A | HIGHLY SIMILAR TO SERINE/THREONINE PROTEIN PHOSPHATASE 2A, 65 KDA REGULATORY SUBUNIT A, ALPHA ISOFORM. |  |  | 3.42 | 2 |
| IPI00217952;IPI00299506 | GFPT1 | ISOFORM OF GLUCOSAMINE--FRUCTOSE-6-PHOSPHATE AMINOTRANSFERASE [ISOMERIZING] 1. |  | 1 | 3.41 | 2 |
| IPI00908469 | TUBB6 | CDNA FLJ52712, HIGHLY SIMILAR TO TUBULIN BETA-6 CHAIN. |  |  | 3.30 | 5 |
| IPI00413324;IPI00478208 | RPL17 | 60S RIBOSOMAL PROTEIN L17; 21 KDA PROTEIN. |  |  | 3.25 | 2 |
| IPI00215911 | APEX1 | DNA-(APURINIC OR APYRIMIDINIC SITE) LYASE. |  | 2 | 3.25 | 1 |
| IPI00003269 | ACTBL2 | BETA-ACTIN-LIKE PROTEIN 2. |  |  | 3.20 | 7 |
| IPI00028635;IPI00383680;IPI00552972 | RPN2 | DOLICHYL-DIPHOSPHOOLIGOSACCHARIDE--PROTEIN GLYCOSYLTRANSFERASE SUBUNIT 2. |  |  | 3.19 | 2 |
| IPI00027192;IPI00943008 | PLOD1 | CDNA, FLJ79184, HIGHLY SIMILAR TO PROCOLLAGEN-LYSINE, 2-OXOGLUTARATE 5-DIOXYGENASE 1. |  |  | 3.18 | 3 |
| IPI00220113;IPI00396171;IPI00878314;IPI00888475 | MAP4 | ISOFORM OF MICROTUBULE-ASSOCIATED PROTEIN 4. |  |  | 3.13 | 2 |
| IPI00015148;IPI00019345;IPI00796349;IPI00816182;IPI00877120;IPI00908754 | RAP1B;RAP1A | RAS-RELATED PROTEIN RAP-1B; RAS-RELATED PROTEIN RAP-1A. |  |  | 3.12 | 2 |
| IPI00221089 | RPS13 | 40S RIBOSOMAL PROTEIN S13. |  |  | 3.12 | 2 |
| IPI00219018 | GAPDH | GLYCERALDEHYDE-3-PHOSPHATE DEHYDROGENASE. |  |  | 3.02 | 23 |
| IPI00744692 | TALDO1 | TRANSALDOLASE. |  |  | 2.98 | 2 |
| IPI00290857;IPI00942512 | KRT3 | KERATIN, TYPE II CYTOSKELETAL 3. |  |  | 2.98 | 2 |
| IPI00176903;IPI00513773;IPI00514023 | PTRF | ISOFORM OF POLYMERASE I AND TRANSCRIPT RELEASE FACTOR. |  |  | 2.95 | 2 |
| IPI00025447;IPI00396485;IPI00472724;IPI00940393 | EEF1A1;EEF1AL3;EEF1A1 | ELONGATION FACTOR 1-ALPHA; ELONGATION FACTOR 1-ALPHA 1; PUTATIVE ELONGATION FACTOR 1-ALPHA-LIKE 3. |  |  | 2.93 | 7 |
| IPI00329389;IPI00790342;IPI00867533 | RPL6 | 60S RIBOSOMAL PROTEIN L6. |  |  | 2.92 | 3 |
| IPI00413344 | CFL2 | COFILIN-2. |  |  | 2.84 | 3 |
| IPI00644127;IPI00910980 | IARS | ISOLEUCYL-TRNA SYNTHETASE, CYTOPLASMIC. |  |  | 2.70 | 2 |
| IPI00005158;IPI00334291;IPI00642982 | LONP1 | LON PROTEASE HOMOLOG, MITOCHONDRIAL. |  |  | 2.67 | 2 |
| IPI00008982;IPI00218547 | ALDH18A1 | ISOFORM OF DELTA-1-PYRROLINE-5-CARBOXYLATE SYNTHETASE. |  |  | 2.62 | 2 |
| IPI00018398 | PSMC3 | 26S PROTEASE REGULATORY SUBUNIT 6A. |  |  | 2.61 | 2 |
| IPI00019472 | SLC1A5 | NEUTRAL AMINO ACID TRANSPORTER B(0). |  |  | 2.59 | 2 |
| IPI00411639;IPI00413108;IPI00553164;IPI00790580;IPI00927101 | RPSAP15;RPSA | LAMININ RECEPTOR-LIKE PROTEIN LAMRL5; 40S RIBOSOMAL PROTEIN SA. |  |  | 2.53 | 3 |
| IPI00941161 | USO1 | GENERAL VESICULAR TRANSPORT FACTOR P115. |  |  | 2.47 | 3 |
| IPI00030275 | TRAP1 | HEAT SHOCK PROTEIN 75 KDA, MITOCHONDRIAL. |  | 1 | 2.43 | 2 |
| IPI00017855;IPI00790739 | ACO2 | ACONITATE HYDRATASE, MITOCHONDRIAL. |  |  | 2.41 | 3 |
| IPI00396485;IPI00472724 | EEF1A1;EEF1AL3;EEF1A1 | ELONGATION FACTOR 1-ALPHA 1; PUTATIVE ELONGATION FACTOR 1-ALPHA-LIKE 3. |  |  | 2.39 | 11 |
| IPI00550746;IPI00871312 | NUDC | NUCLEAR MIGRATION PROTEIN NUDC. |  |  | 2.35 | 2 |
| IPI00383581;IPI00441414 | GANAB | CDNA FLJ61290, HIGHLY SIMILAR TO NEUTRAL ALPHA-GLUCOSIDASE AB. |  |  | 2.31 | 3 |
| IPI00027223;IPI00925000 | IDH1 | ISOCITRATE DEHYDROGENASE [NADP] CYTOPLASMIC. |  |  | 2.31 | 2 |
| IPI00004860;IPI00759723 | RARS | ISOFORM OF ARGINYL-TRNA SYNTHETASE, CYTOPLASMIC. |  |  | 2.31 | 2 |
| IPI00031169;IPI00873632 | RAB2A | RAS-RELATED PROTEIN RAB-2A. |  |  | 2.26 | 2 |
| IPI00257508 | DPYSL2 | DIHYDROPYRIMIDINASE-RELATED PROTEIN 2. |  |  | 2.24 | 3 |
| IPI00009236;IPI00759683;IPI00852906;IPI00853146;IPI00876941 | CAV1 | CAVEOLIN. |  |  | 2.13 | 2 |
| IPI00029601;IPI00062884;IPI00746806 | CTTN | SRC SUBSTRATE CORTACTIN. |  | 1 | 2.12 | 1 |
| IPI00100160;IPI00604431 | CAND1 | ISOFORM 1 OF CULLIN-ASSOCIATED NEDD8-DISSOCIATED PROTEIN 1. |  |  | 2.11 | 2 |
| IPI00056334 | PRKCDBP | PROTEIN KINASE C DELTA-BINDING PROTEIN. |  |  | 2.08 | 1 |
| IPI00106642;IPI00257508 | SDF2L1;DPYSL2 | DIHYDROPYRIMIDINASE-LIKE 2. |  |  | 2.08 | 3 |
| IPI00746165;IPI00873622 | WDR1 | ISOFORM 1 OF WD REPEAT-CONTAINING PROTEIN 1. |  |  | 2.07 | 2 |
| IPI00023122;IPI00023560 | PDLIM7 | ISOFORM OF PDZ AND LIM DOMAIN PROTEIN 7. |  |  | 2.05 | 2 |
| IPI00007074 | YARS | TYROSYL-TRNA SYNTHETASE, CYTOPLASMIC. |  |  | 2.05 | 2 |
| IPI00306825;IPI00399265;IPI00399267;IPI00743469 | TPD52L2 | ISOFORM OF TUMOR PROTEIN D54. |  |  | 1.96 | 3 |
| IPI00012490;IPI00021695;IPI00216526;IPI00216527;IPI00216528;IPI00216529;IPI00216530;IPI00217164;IPI00217165;IPI00217166;IPI00217168;IPI00217169;IPI00217170;IPI00217171 | ATP2B4;ATP2B1 | ISOFORM XD OF PLASMA MEMBRANE CALCIUM-TRANSPORTING ATPASE 4. |  |  | 1.89 | 3 |
| IPI00396171;IPI00878314;IPI00888475 | MAP4 | ISOFORM OF MICROTUBULE-ASSOCIATED PROTEIN 4. |  |  | 1.88 | 3 |
| IPI00783271 | LRPPRC | LEUCINE-RICH PPR MOTIF-CONTAINING PROTEIN, MITOCHONDRIAL. |  |  | 1.80 | 2 |
| IPI00002606;IPI00044745;IPI00879729 | SCIN | ISOFORM OF ADSEVERIN. |  |  | 1.75 | 3 |
| IPI00015262;IPI00398735;IPI00910593 | CNN2 | CALPONIN-2. |  |  | 1.73 | 2 |
| IPI00002520;IPI00748411;IPI00794549;IPI00794572;IPI00908824;IPI00909681 | SHMT2 | SERINE HYDROXYMETHYLTRANSFERASE, MITOCHONDRIAL. |  |  | 1.71 | 2 |
| IPI00018931;IPI00943562 | VPS35;LOC100133770 | VACUOLAR PROTEIN SORTING-ASSOCIATED PROTEIN 35. |  |  | 1.69 | 2 |
| IPI00016339 | RAB5C | RAS-RELATED PROTEIN RAB-5C. |  |  | 1.68 | 2 |
| IPI00289499;IPI00925601 | ATIC | BIFUNCTIONAL PURINE BIOSYNTHESIS PROTEIN PURH. |  | 1 | 1.67 | 4 |
| IPI00215965;IPI00465365;IPI00644968;IPI00760620;IPI00797148;IPI00879501;IPI00879518 | HNRNPA1;HNRPA1L3;HNRPA1L-2;HNRNPA1L2 | ISOFORM A1-B OF HETEROGENEOUS NUCLEAR RIBONUCLEOPROTEIN A1; ISOFORM A1-A OF HETEROGENEOUS NUCLEAR RIBONUCLEOPROTEIN A1; PUTATIVE HETEROGENEOUS NUCLEAR RIBONUCLEOPROTEIN A1-LIKE PROTEIN 3; SIMILAR TO HETEROGENEOUS NUCLEAR RIBONUCLEOPROTEIN A1; ISOFORM 2 OF HETEROGENEOUS NUCLEAR RIBONUCLEOPROTEIN A1; HETEROGENEOUS NUCLEAR RIBONUCLEOPROTEIN A1-LIKE PROTEIN 2; 29 KDA PROTEIN. |  |  | 1.63 | 6 |
| IPI00294834;IPI00944640 | ASPH | ASPARTYL/ASPARAGINYL BETA-HYDROXYLASE. |  |  | 1.54 | 2 |
